# Concurrent HIIT and Resistance Training for Musculoskeletal Function: A Systematic Review of Neuromuscular, Morphological, and Performance Adaptations

**DOI:** 10.3390/life16030381

**Published:** 2026-02-27

**Authors:** YuWei Chang, Hsia-Ling Tai, Cheng-Long Yang, Chun-Hsien Su

**Affiliations:** 1 Graduate Institute of Sports Training, University of Taipei, Tian-Mu Campus, Taipei City 111036, Taiwan; d10935006@go.utaipei.edu.tw; 2 Department of Physical Education, University of Taipei, Bo-Ai Campus, Taipei City 110234, Taiwan; danatai1008@go.utaipei.edu.tw; 3 Department of Interdisciplinary Baccalaureate Degree Program, Chinese Culture University, Taipei City 111396, Taiwan; norman@ulive.pccu.edu.tw; 4 Department of Physical Education, Putian University, Putian 351100, China; 5 Department of Exercise and Health Promotion, Chinese Culture University, Taipei City 111396, Taiwan

**Keywords:** concurrent training, high-intensity interval training, neuromuscular adaptation, muscle architecture, tendon, strength and power, older adults, athletes

## Abstract

This systematic review focuses on the effect of concurrent high-intensity interval training (HIIT) and resistance training on musculoskeletal function in adult individuals. Four electronic databases (PubMed, Web of Science Core Collection, Scopus, and PsycINFO) were searched for controlled trials in older or middle-aged adults, in recreationally exercising adults, and in athletic or tactical populations, which completed parallel HIIT and resistance training and described musculoskeletal responses to the intervention up to 30 November 2025. A total of 18 trials fulfilled the eligibility criterion and were synthesized narratively across the domains of maximal strength, explosive performance, neuromuscular activity, muscle morphology and architecture, tendon-related outcomes, and adherence and safety. Most 8- to 12-week interventions maintained two to three weekly resistance sessions and were designed in time-effective HIIT formats, increasing or preserving maximal strength in older subjects as well as younger ones that were trained. Explosive performance metrics, including both jump and sprint tasks, were usually preserved or even improved by the maintenance of the power-oriented component in resistance-based exercise sessions. The limited electromyography data indicated improved neuromuscular activation during submaximal tasks, particularly in older subjects, whereas some studies reported subtle increases or maintenance of muscle size and selective architectural patterns during application of progressive loading. Tendon-specific adaptations are difficult to measure, as imaging was seldom available, but functional tasks influenced by the muscle–tendon unit have been studied in multiple studies. Adherence was good, and adverse events were rare in all studies. Overall, the evidence suggests that well-designed concurrent HIIT and resistance training programs can improve or maintain musculoskeletal performance, although the magnitude and expression of these adaptations vary according to population characteristics and intervention design. Importantly, by integrating neuromuscular, morphological, and performance-related outcomes across diverse adult populations, this review provides a musculoskeletal-centered synthesis that extends prior concurrent training reviews beyond cardiorespiratory or interference-focused perspectives.

## 1. Introduction

### 1.1. Musculoskeletal Function and Integrative Assessment

Musculoskeletal function represents the coordinated interaction of neural input, muscle contractile elements, connective tissues, and joint mechanics. Effective movement depends on how the nervous system regulates motor unit recruitment, firing frequency, and synchronization, all of which shape the magnitude and rate of force generation [1,2,3]. Structural factors such as muscle fiber pennation angle, fascicle length, and tendon stiffness further influence the efficiency of force transmission and movement economy [4,5,6]. These properties respond to mechanical loading in different ways, contributing to training-induced changes in performance and movement quality.

Improvements in measurement tools have supported a more comprehensive understanding of musculoskeletal processes. Surface electromyography and isokinetic dynamometry are commonly used to assess neural activation strategies and joint torque or power across movement velocities [7]. In addition, ultrasonography and magnetic resonance–based techniques enable noninvasive evaluation of muscle architecture, tendon-related characteristics, muscle volume, and metabolic responses and are sensitive to training-induced structural adaptations [8,9]. Collectively, these measurement approaches support a multidimensional assessment of musculoskeletal adaptation across neural, structural, and functional domains.

In this review, the term “muscle architecture” is used to describe structural properties of skeletal muscle (e.g., fascicle length and pennation angle), whereas “neuromuscular efficiency” refers to neural activation and coordination characteristics underlying force production. Musculoskeletal performance is used as a higher-order functional construct encompassing strength, power, and task-specific performance outcomes arising from the integration of neural, muscular, and structural factors.

### 1.2. HIIT, Resistance Training, and Neuromuscular Adaptations

HIIT is well defined as one of the most impactful measures for the improvement of cardiorespiratory fitness over shorter training periods [10]. HIIT is believed to also influence neuromuscular activation, movement efficiency, and muscular power index by motor unit activation and fatigue resistance [11,12]. On the contrary, HIIT tends to achieve only modest increases in maximal strength or muscle hypertrophy as it focuses on metabolic rather than mechanical loading.

The primary stimulus that drives improved maximal force, muscle hypertrophy, tendon stiffness, and neuromuscular efficiency in most cases remains resistance training [13,14,15,16]. Mechanotransduction pathways such as mTOR signaling, satellite cell activity, and structural remodeling are well-suited to the enhancement of musculoskeletal properties associated with strength and power [13,14,15,16]. The combination of HIIT and resistance exercise may enhance, through the provision of concurrent metabolic and mechanical stimuli, the corresponding training modes. Such integrative potential was emphasized in our previous narrative review that summarized possible mechanisms by which HIIT and resistance training jointly influence neuromuscular and morphological outcomes [17].

In this review, concurrent HIIT and resistance training are defined as programs that intentionally mix high-intensity interval training with resistance or power-based exercises within the same overall intervention. Such concurrent forms are applied in three main populations that the present review will focus on: older and middle-aged adults, recreationally active adults, and athletic or tactical populations.

Although narrative work has provided mechanistic knowledge about how these combined approaches affect musculoskeletal outcomes, the reviews cannot ascertain if their effect is consistent, nor can the impacts of training variables (e.g., weekly frequency, session sequencing, or exercise intensity) be elucidated. Such limitations highlight the necessity to investigate concomitant HIIT and resistance training on musculoskeletal adaptations.

### 1.3. Rationale for a Systematic Review on Concurrent HIIT and Resistance Training

While concurrent training studies have existed for quite a while, most studies revolve around endurance–strength interactions or athletic performance, and little research has approached musculoskeletal function as a multidimensional construct [18]. Evidence on concurrent HIIT and resistance training is found in older and middle-aged adults, recreationally active adults, and athletic or tactical populations, and diverse protocols are applied. This dispersion also makes it difficult to measure if certain combinations of HIIT and resistance loading consistently produce enhanced strength, power, neuromuscular activation, or muscle morphology. Some research may indicate either that sequencing HIIT before resistance exercise might influence acute molecular signaling or that such an alternation, or separation of sessions, is effective to mitigate potential interference effects [19,20]. These results are not consistent and have seldom been investigated within a single framework targeting musculoskeletal outcomes.

A systematic review is therefore warranted for several reasons. Musculoskeletal function encompasses neural, structural, and functional domains, and understanding the effects of concurrent HIIT and resistance training requires integrated evaluation of strength, power, neuromuscular activation, and morphological outcomes. In addition, older adults, recreationally active individuals, and athletic or tactical populations differ substantially in baseline characteristics, training objectives, and adaptive priorities, which are likely to shape their responses to concurrent training. Despite this, no previous synthesis has examined these populations collectively with a primary focus on musculoskeletal adaptations. Furthermore, considerable methodological variation across trials, including differences in interval formats, resistance training volume, and session sequencing, may meaningfully influence adaptation patterns, underscoring the need for a transparent and systematic assessment.

In contrast to previous narrative work, the present review applies explicit eligibility criteria, a standardized search strategy, and a formal risk of bias evaluation [18,19,20]. This approach enables a structured and transparent synthesis of the effects of concurrent HIIT and resistance training on musculoskeletal performance across diverse adult populations.

Although concurrent training has been extensively examined from cardiorespiratory and interference-oriented perspectives, musculoskeletal function has seldom been addressed as an integrated outcome spanning neuromuscular, morphological, and performance-related domains. Accordingly, the present systematic review adopts a musculoskeletal-centered framework to synthesize evidence on adaptations to concurrent HIIT and resistance training. By systematically examining controlled trials in older adults, recreationally active individuals, and athletic or tactical populations, this review aims to clarify not only whether concurrent training is compatible with musculoskeletal development but also how population characteristics and training design features shape adaptive responses. This integrative perspective is intended to advance current understanding beyond cardiorespiratory or interference-focused interpretations of concurrent training and to support population-specific interpretation rather than implying a uniform response across all adult groups.

### 1.4. Objectives

The current review was developed for three main objectives. The first aim was to collate controlled trials examining how concurrent HIIT and resistance training affect musculoskeletal performance in healthy adults and athletes. The second aim was to characterize the associated adaptations in maximal strength, power, neuromuscular activation, muscle architecture, and tendon-related characteristics. The third aim was to investigate whether variations in training structure, such as same-day versus alternating-day scheduling or the order in which HIIT and resistance training are performed, are associated with different adaptation patterns. Collectively, these aims are intended to provide an evidence-based framework for interpreting how integrative training methods influence musculoskeletal function. By synthesizing evidence on concurrent HIIT and resistance training, this review directly addresses how integrative exercise modalities shape muscle function across neural, structural, and performance domains, in alignment with the scope of the present Special Issue on exercise training and muscle function.

## 2. Methods

### 2.1. Protocol and Reporting

The review protocol, including the research question, eligibility criteria, and planned analytical procedures, was specified in advance. The review protocol was not prospectively registered in PROSPERO or any other public registry. Nevertheless, the research questions, eligibility criteria, and planned analytical procedures were specified in advance, and the conduct and reporting of the review followed contemporary recommendations for transparent methodology in exercise and health sciences. Reporting was structured in accordance with the Preferred Reporting Items for Systematic Reviews and Meta-Analyses (PRISMA) 2020 statement [21], and we drew on additional guidance regarding documentation of database searches and reproducibility when designing the search strategy and mapping the study selection process [22]. The review focused on controlled trials that implemented concurrent HIIT combined with resistance training and reported musculoskeletal outcomes in healthy adults or athletic populations. For the purposes of this review, HIIT was used as an umbrella term encompassing short-duration, high-intensity interval-based endurance protocols, including sprint interval training (SIT) and repeated sprint training, provided that the training intensity and work-to-rest structure met conventional definitions of HIIT. Terminology is applied consistently throughout the manuscript according to this framework.

### 2.2. Data Sources and Search Strategy

A structured search strategy was employed to identify controlled trials investigating the effects of concurrent HIIT and resistance training on musculoskeletal outcomes in healthy adults and athletic populations. Four electronic databases were selected because they collectively cover exercise physiology, biomedical research, and behavioral science: PubMed, Web of Science Core Collection, Scopus, and PsycINFO (EBSCO). These sources encompass a broad range of journals in kinesiology, physiology, sports science, rehabilitation, and applied health.

The search strategy combined controlled vocabulary and free-text terms related to concurrent training, interval training, resistance exercise, musculoskeletal function, and neuromuscular or morphological adaptations. Boolean operators (“AND”, “OR”) were used to structure the search strings, which were adapted to the indexing and syntax requirements of each database. No limits on study design, population, or publication type were applied at the search stage to minimize the risk of missing relevant trials. The strategy was developed and refined through pilot searches until it consistently retrieved several prespecified key articles. Its design was informed by recommendations for transparent reporting in systematic reviews [21] and by guidelines for documenting literature searches [22]. Full, database-specific search strings are reported in Supplementary Table S1.

All database searches were completed up to 30 November 2025, with no restriction on publication year. Only articles published in English were considered. To complement the electronic searches, reference lists of eligible trials and relevant reviews were screened manually to identify additional studies that might not have been captured in the database queries.

### 2.3. Eligibility Criteria (PICO Framework)

The eligibility criteria were set by a Population–Intervention–Comparison–Outcome (PICO) approach consisting of studies that investigated musculoskeletal responses to combined HIIT and resistance training.

Population (P). We included studies of healthy adults spanning the lifespan and covering recreationally active, trained, athletic, and tactical populations. Participants were required to be free from diagnosed cardiometabolic, neuromuscular, or orthopedic disease. Trials of clinical populations (cardiometabolic or orthopedic conditions), adolescents, and patients who were recovering from injury or surgery were excluded in order to minimize physiological heterogeneity and direct findings towards healthier adult or athletic responses.

Intervention (I). In eligible trials, concurrent training protocols in which HIIT and resistance exercise were performed as part of the intervention, either within the same session or on distinct training days within the same program, were used. Other interventions not involving an actual concurrent structure or that combined resistance training with only steady-state aerobic exercise were excluded.

Comparison (C). The effect of the concurrent training was considered to be isolated in the trials if at least one comparator condition (e.g., resistance-only training, HIIT-only training, traditional endurance training, or an alternative sequencing or configuration of HIIT and resistance sessions) was included. Studies without a distinguishable control or comparison group were excluded.

Outcomes (O). The studies had to report at least one musculoskeletal outcome as assessed by established physiological or biomechanical methods. Relevant domains were maximal strength, power, neuromuscular activation, muscle morphology, and tendon properties. Outcomes may be evaluated by means of dynamometry, electromyography, ultrasound, magnetic resonance imaging, or standardized performance tests. We excluded studies reporting the absence of pre–post musculoskeletal changes or only non-musculoskeletal outcomes.

Although eligibility criteria were primarily structured using a PICO framework, they are fully compatible with a PECOS approach, with the intervention conceptualized as a structured exercise exposure and study design explicitly specified.

Additional criteria. We focused on peer-reviewed, full-length articles in English. Conference abstracts, dissertations, study protocols, commentaries, and narrative reviews were not included. Trials with insufficient methodological detail for interpretation (i.e., incomplete description of training protocols or outcome measures) and those without quantifiable pre- and post-data were also excluded to ensure that there was a consistent, methodologically rigorous source for assessing musculoskeletal responses to concurrent HIIT and resistance training in healthy adult subjects.

Studies were excluded only when musculoskeletal outcomes were not reported in a manner permitting pre–post comparison or quantitative interpretation, irrespective of whether the results were positive, null, or unfavorable.

### 2.4. Study Selection

Study selection used the same structured, sequential approach as per published guidelines for systematic reviews. The search of databases published via PubMed, Web of Science, Scopus, and PsycINFO yielded 1416 records, which were imported into a reference management system. We identified 474 duplicates through automated as well as manual techniques and left 942 novel records for screening. Two reviewers screened titles and abstracts independently to determine if each record described concurrent HIIT combined with resistance exercise and reported at least one musculoskeletal outcome. For the specific reasons above, 867 records were excluded at this point in time, and 75 articles were continued for full-text evaluation.

**Figure 1:**
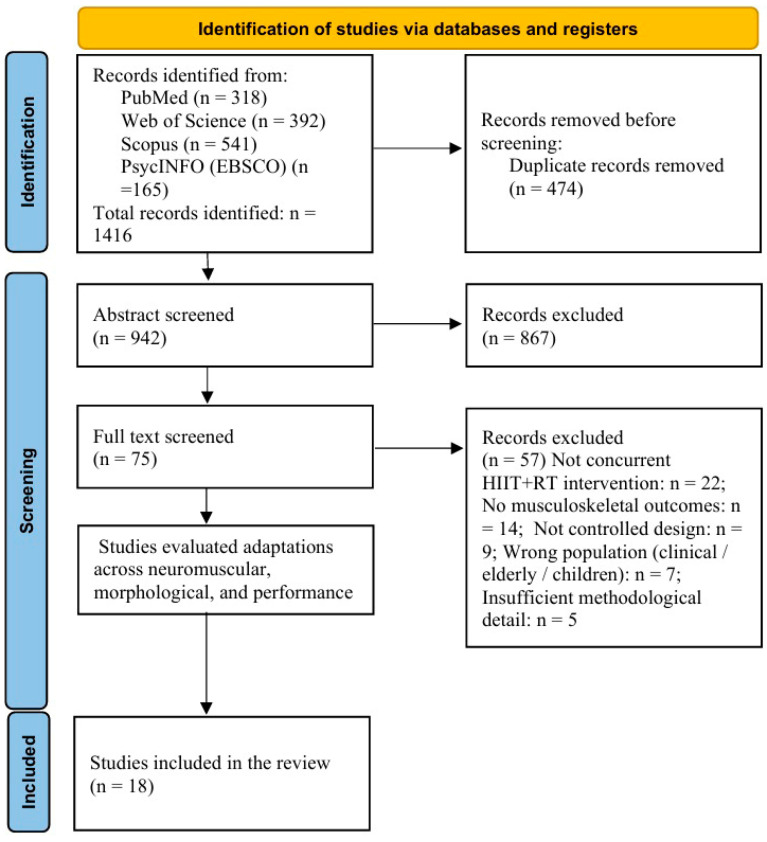
PRISMA 2020 flow diagram summarizing the identification, screening, eligibility assessment, and final inclusion of controlled trials evaluating the effects of concurrent high-intensity interval training and resistance training on musculoskeletal outcomes in healthy adults and athletic populations.

Full-text screening was conducted to assess study aims, participant characteristics, intervention structure, and outcome measures. We excluded studies that did not carry out true concurrent HIIT plus resistance training, recruited ineligible clinical or age-defined populations, did not provide relevant musculoskeletal outcomes, or did not offer adequate methodological detail for interpretation. After this procedure, 57 full-text publications were excluded, and 18 controlled trials were deemed eligible for the final synthesis. The title, abstract, and full-text disagreements were debated and resolved by discussion to ensure that all eligibility criteria were applied consistently. The PRISMA flow diagram (Figure 1) shows the distribution of records in identification, screening, eligibility, and inclusion. A PRISMA-compliant summary of the 57 full-text exclusions according to the reasons mentioned above is given in Supplementary Table S2, and a study-level exclusion log is included in Supplementary Table S2a.

### 2.5. Data Extraction

Data extraction was conducted according to a structured template in order to capture key characteristics of each trial and to maintain consistency in data handling. For each study, we collected sample size, participant demographics, baseline training status, and any information on sport specialization or performance level. We tracked total program duration, weekly training frequency, the structure and sequencing of HIIT and resistance sessions, and the characteristics of the comparison condition or conditions.

Musculoskeletal outcomes were obtained in their original units and formats, including measures of maximal strength, power, neuromuscular activation, muscle morphology, tendon properties, and functional performance tests. Pre- and post-intervention means, standard deviations, change scores, and reported effect sizes were extracted when available. Compliance-related data, including session attendance and reported adverse events, were also extracted and reported, as summarized in Supplementary Table S3.

One reviewer processed all extraction fields and independently cross-checked them by a second reviewer. Discrepancies or ambiguities in the extracted data were discussed to resolve any inconsistencies with reference to the original article.

### 2.6. Data Synthesis

Considering the heterogeneity across the included trials about training setups, participant characteristics, outcome measures, and reporting formats, a narrative synthesis was the primary method of evidence integration. Quantitative meta-analysis was not performed since the wide range of protocols, measurement tools, and time frames impeded the feasibility and interpretability of pooled effect estimates.

Heterogeneity was evident a priori across multiple dimensions, including participant characteristics (age, training status), intervention design (HIIT format, resistance training volume and intensity, session sequencing), outcome domains (strength, power, neuromuscular activation, muscle architecture, tendon-related measures), and measurement methods. Under these conditions, the calculation of pooled effect sizes and heterogeneity statistics (e.g., I^2^ or Cochran’s Q) was not methodologically appropriate, as outcomes were not sufficiently comparable to support meaningful quantitative synthesis. Similarly, subgroup analyses were not attempted because the number of studies within specific outcome domains and population strata was insufficient to support statistically robust or interpretable subgroup comparisons. Accordingly, a structured narrative synthesis was adopted to allow outcome-specific and population-specific interpretation while preserving methodological transparency.

Findings were categorized thematically, with the respective musculoskeletal functions reflected in main themes (strength and power outcomes, neuromuscular and electrophysiological indicators, morphological adaptations of muscle and tendon, and performance-based functional outcomes). Within these domains, we compared results across trials and observed similarities and differences between responses to concurrent HIIT and resistance training relative to comparator conditions. Where feasible, we considered not only the direction and magnitude of changes but also contextual features such as training status, program duration, and the sequencing or distribution of HIIT and resistance sessions. A detailed summary of outcome domains and principal measures per trial is provided in Supplementary Table S4. This systematic review was conducted and reported in accordance with the PRISMA 2020 guidelines (see Table S5 in the Supplementary Materials).

### 2.7. Risk of Bias Assessment

The risk of bias was assessed independently by two reviewers using the Cochrane risk of bias tool for randomized trials. The following domains were considered: sequence generation, allocation concealment, blinding of participants, personnel, and outcome assessors, completeness of outcome data, selective reporting, and other potential threats to internal validity. “Unclear” judgments on a specific domain were noted when information for that domain was not clearly described, with a rationale to support this judgment documented.

Discrepancies over risk-of-bias judgments were resolved by discussion to a consensus level. Overall risk of bias ratings per study are presented in Table 1, with item-level assessments and justification in Supplementary Table S6. Instead of considering such evaluations as a reason for exclusion of studies from the synthesis, these evaluations were used to help assess the strength and confidence of the evidence as an index of comparisons across trials.

## 3. Results

Across the included studies, substantial heterogeneity was observed in participant characteristics, training protocols, intervention duration, and outcome measures. Study populations ranged from older and middle-aged adults to recreationally active individuals and athletic or tactical populations, with differing baseline fitness levels and training objectives. Intervention durations typically ranged from 8 to 12 weeks, although session frequency, resistance training volume, and the structure and intensity of HIIT varied considerably between trials. Outcome assessments encompassed maximal strength, explosive performance, neuromuscular activity, and muscle morphology, with variability in testing modalities and reporting approaches. This heterogeneity is considered in the interpretation of results across outcome domains in the following sections.

### 3.1. Included Studies

The qualitative synthesis included 18 trials in total. The process of study selection is summarized in Figure 1. Searching databases, 1416 records were retrieved and entered into the reference management system for screening. Following duplicate removal, the remaining records underwent title and abstract screening; however, most were excluded because they did not investigate concurrent HIIT and resistance training or did not report musculoskeletal outcomes. Full texts were retrieved for 75 articles, 57 of which were excluded after a strict eligibility review. The remaining 18 studies met all their inclusion criteria and were retained for final synthesis.

### 3.2. Overview of Included Studies

The 18 included trials [23,24,25,26,27,28,29,30,31,32,33,34,35,36,37,38,39,40] involved a variety of populations, training designs, and outcome domains. The participation size varied from small laboratory-based trials to comprehensive community- or team-based interventions, ranging from 14 participants to 120. Six trials included older or middle-aged adults [23,24,31,32,36,38], and the rest recruited trained athletes, recreationally active adults, or physically trained men and women.

The training duration varied from very short 2-week protocols to more conventional 8–12-week interventions [23,24,25,26,27,30,31,32,33,34,35,36,38,39,40]. Most studies used a HIIT format, which featured repeated 15–60 s bouts performed at an intensity in the ballpark of 90–120% maximal aerobic power, combined with resistance and power-specific tasks targeting large lower limb muscle groups. Training frequency ranged from 2 to 4 sessions per week, and some athlete-centered investigations incorporated pre-season or in-season variance of load distribution [33,34,35].

The outcome domains were maximal dynamic or isometric strength, countermovement jump performance, sprint ability, aerobic capacity, muscle morphology, tendon-related measurements, and electromyographic indices, as well as acute molecular or biochemical markers [27,37,38,39,40]. Studies using older adults focused mainly on functional outcomes and lean mass preservation, while studies in sports cohorts emphasized explosive performance, repeated sprint ability, and sport-specific neuromuscular metrics. The main characteristics of the sample and training are summarized in Table 2; musculoskeletal outcome domains and primary measures are listed in Supplementary Table S4.

### 3.3. Effects on Maximal Strength

In the included trials [23,24,25,26,27,28,29,30,31,32,33,34,35,36,37,38,39,40], concurrent HIIT and resistance training generally produced favorable but heterogeneous effects on maximal strength. Improvements were reported in both upper and lower limb strength, although the magnitude of change differed by program duration, baseline training status, and the relative emphasis placed on heavy versus power-oriented resistance loading.

In older and middle-aged adults, 8- to 12-week interventions resulted in small to moderate improvements in leg press, knee extension, and global functional strength [23,24,31,32]. In recreationally active adults and in athletic or tactical populations, concurrent programs usually increased one repetition maximum values in major multi-joint lifts or sport-specific strength tasks when resistance loading was maintained at moderate to high levels [25,29,33,39,40]. These adaptations were noted in a variety of HIIT formats, such as running intervals, cycling intervals, and sport-specific repeated sprint protocols.

The effects of shorter programs were more variable. Two-week interventions that combined all-out sprints with resistance training did not consistently improve maximal strength [26,27]. In contrast, several pre-season or in-season studies in athletes reported that four- to six-week concurrent blocks could at least maintain, and in some cases modestly improve, maximal force output when resistance sessions were clearly included in the weekly schedule [28,33,34,35].

Altogether, we conclude that maximal strength can be sustained or enhanced using concurrent HIIT and resistance training if the resistance loading is retained within the structure of the program. In contrast, very short or metabolically dominant protocols tend to show fewer effects.

### 3.4. Effects on Power and Explosive Performance

Performance of simultaneous HIIT and resistance training was relatively unchanged or changed the outcomes on power-related tasks, but the response to such studies by population and program type was variable. Relevant measures for things such as countermovement jump height, squat jump, short sprint times, and change-direction showed evidence of positive changes in large parts of the interventions with resistance working hard and high velocity or power-based movements.

In athletic and tactical groups, pre-season and in-season programs incorporating repeated sprint and short interval running, coupled with lower body strength and power exercises, contributed to or provided maintenance (improvement or at least the same level of development) of jump performance and sprint capacity over four- to eight-week periods [28,29,33,34,35,39,40]. The younger players in soccer and rugby, for instance, improved or retained their countermovement jump height and repeated sprint performance, as well as 10–30 m sprint times after an additional high-intensity running volume [28,33,34,35]. Similarly, elite water polo athletes reported that simultaneous interval and strength training was associated with enhanced repeated sprint swimming performance [29].

In older and middle-aged adults, concurrent activities resulted in small to moderate improvement in functional power tasks (e.g., chair rise tests, rapid stepping, and stair climbing) over 8-to-12-week interventions [23,24,31,32]. Fluctuations in jump height or maximal sprint velocity were less commonly documented in these cohorts, but improvements in functional explosive tasks indicated meaningful improvement in daily life performance.

The results for very short-duration protocols were mixed. It was observed that the two-week sprint interval plus resistance interventions did not improve vertical jump or peak power outcomes consistently [26,27]. Meanwhile, trials that specifically included explosive or ballistic resistance training were more likely to report improved jump performance and rate of force development, particularly among younger and/or trained persons [25,36,37,38].

Collectively, the findings suggest that explosive performance is frequently maintained or enhanced when HIIT and resistance training are combined with a specific power-focused resistance part and when program duration and loading are adequate.

### 3.5. Effects on Neuromuscular Activity (EMG)

Surface electromyography results were indicated only in a handful of trials, and EMG collection and analysis protocols varied widely with respect to results. Despite the heterogeneity, distinct descriptive patterns emerge when EMG measurements are interpreted in conjunction with changes in strength, power, and functional capacity.

In older and middle-aged individuals, pre- and post-training and submaximal task analyses and EMG amplitudes were either unchanged or reduced to a modest extent after the concurrent HIIT and resistance programs in studies of submaximal performance in this age group [23,24,30,31]. These alterations also accompanied improvements in strength and functional tests, including chair rise performance, gait speed, and balance [23,24,30,31]. Consequently, participants were able to do the same (or more) external work while experiencing the same (or less) documented muscle activation.

EMG-derived indices exhibited task-specific responses in recreationally active adults and athletic or tactical populations. Despite the retention of high-intent resistance exercises and moderate HIIT volume, the activation of explosive or high-velocity movements, even in the intense phases of the training process, remained constant or slightly increased [26,27,28,29,39,40]. As such, these EMG findings were accompanied by improvements or maintenance of jump and sprint performance in multiple trials under concurrent training structures [41].

Overall, across the interventions that included EMG, a few were able to suggest significant increases in maximal strength or power that were accompanied by little or no increase in amplitude of EMG. Due to the small samples and high methodological variation in EMG procedures [40,41], these neutral findings do not permit definitive conclusions concerning the level of neural adaptation. Despite this, in contrast to studies of performance results, the data can show that concurrent HIIT and resistance training can potentially retain the neuromuscular activation patterns used in the tasks being assessed.

### 3.6. Effects on Muscle Morphology and Architecture

Morphological findings were presented only in a sample of the studies [23,24,25,26,27,28,29,30,31,32,33,34,35,36,37,38,39,40], and the degree of structural adaptation was dependent on age, training status, and program design. We observed and reported measures of muscle cross-sectional area, muscle thickness, and fiber-specific hypertrophy, as well as architectural parameters such as pennation angle and fascicle length.

In older and middle-aged individuals, 8-to-12-week interventions that included HIIT with traditional or power-oriented resistance training induced small increases or maintenance in the muscle size indices (i.e., quadriceps thickness, thigh lean tissue, or whole leg muscle mass) [23,24,31,32]. Although the structural changes in those programs were relatively small compared to traditional training models, they often made an enormous difference in muscle strength and performance [42].

Several concurrent sets of programs provoked more significant changes in muscle structure in recreationally active adults, as well as men and women exercising or athletes in combat teams. The increasing presence and progressive progression of resistance and the accompanying short-term HIIT and sprint intervals on the same regimen led to an increase in muscle thickness, cross-sectional area of fibers, and pennation angle [26,27,28,29,39,40]. Modifications in muscle shape, as with increased pennation angle or preserved fascicle length, were detected in some studies, which correlated with improvements in strength, power, and sprint performance [25,36,37,38,43].

Quantitative morphological alteration was not achieved across all regimens. Interventions providing very brief, repetitive sprints or all-out efforts with little to no resistance volume did not significantly increase muscle thickness or mass [26,27]. Similarly, a few youth interventions within team sports that targeted technical and aerobic training in the same format only revealed marginal change in muscle size with an improvement in performance [28,33,34].

Taken together, the results show that simultaneous HIIT and resistance training can maintain or enhance muscle size and the selected architecture attributes when resistance loading and program duration are adequate, particularly in younger or trained adults. Structural adaptations have a lesser effect in older adults but exist alongside significant improvements in strength and function.

### 3.7. Effects on Tendon Properties

Tendon characteristics and mechanical properties assessed directly were largely missing from the concurrent training trials. None of these studies showed ultrasound evaluation of tendon stiffness, cross-sectional area, or elongation under load, and no protocol provided MRI or elastography findings specific to tendon tissue.

In older and middle-aged adults, functional tasks involving the muscle–tendon unit, such as rapid sit-to-stand transitions, stepping, or power-based lower limb tasks, included in several of the interventions reported improvements [23,24,31,32]. Among recreationally active and athletic or tactical populations, gains in countermovement jump performance, sprint ability, and repeated high-intensity efforts were also apparent [28,29,33,34,35,38,39]. Yet, in the absence of specific tendon measurements, the changes cannot be ascribed to individual differences in tendon characteristics.

Therefore, the extent of adaptations to concurrent HIIT and resistance training at the tendon level is unclear in the present evidence, and any changes in performance need to be explained by reference to tendon behavior.

### 3.8. Adherence and Adverse Events

In the included trials, adherence was usually at a high percentage of simultaneous high-intensity interval and resistance training, with most reports indicating session attendance in the range of 85–95 percent [23,24,25,26,27,28,29,30,31,32,33,34,35,36,37,38,39,40]. The highest adherence is most likely to be evident in controlled laboratory interventions and in groups where interventions were implemented in conjunction with pre-season or ongoing training programming, especially in athletic populations, where training programs were introduced to pre-season or in-season training programs [28,29,33,34,35]. Several interventions among older adults reported nearly full participation over eight to twelve weeks of training [23,24,31,32].

Adverse events were uncommon. In most trials, there were no injuries, medical complications, or loss of training, explained by the simultaneous treatments [23,24,29,31,32,33,38,39]. The most often reported response was mild, transient muscle soreness. Even following sprint-based protocols or repeated-effort efforts, no study reported performance-limiting fatigue or overuse symptoms causing withdrawal from training [26,27,28,34,35]. Supplementary Table S3 provides the study-specific adherence data and adverse event reports.

### 3.9. Summary of Main Findings from the Included Evidence

The 18 trials evaluated suggest that combining concurrent HIIT and resistance training can enhance multiple aspects of musculoskeletal performance in both healthy adults and athletes. Maximal strength was found to be increased when resistance training was subjected to an adequate mechanical load independently of whether the concurrent aerobic stimulus was delivered as running-based HIIT, cycling intervals, or sport-specific repeated sprint formats. The strength gains were seen across age groups and were similar to those with the resistance-only protocols of equivalent duration.

Explosive performance (i.e., jump height, sprint ability, and response to rapid force tasks) was also enhanced in many interventions, especially with young sporting adults in high-velocity or power-based resistance training interventions. The direction of effect differed, but the overall phenomenon is that HIIT did not affect an absolute decrease in jump or sprint performance when added to appropriate programs.

Neuromuscular results were favorable in terms of movement speed, force rate, and resistance to fatigue with repeated efforts, as reported in the study in the last analysis. Morphological measures such as muscle thickness, cross-sectional area, and some selected architectural features showed a greater improvement in several longer-duration interventions that involved progressive loading with resistance, while shorter or metabolically oriented protocols suggested more subtle structural change.

It was not directly quantified whether the tendon benefited individually or collectively, and any tendon-level adaptability can only be inferred based on performance measures that utilize the muscle-tendon unit. Adherence was high and adverse events were rare across studies, so the concurrent programs considered in this review were viable to implement in older and younger adult populations.

Collectively, these trials have suggested that concurrent HIIT and resistance training can preserve or ameliorate strength, power, and functional outcomes in healthy adults. Differences in the outcome appear to be substantially dictated by program duration, resistance/HIIT balance, and participants’ characteristics. The musculoskeletal results based on the population group are summarized in Table 3, and training design features related to beneficial vs. non-supportive adaptability are presented in Table 4.

## 4. Discussion

Rather than reflecting a uniform response, the findings of this review indicate that musculoskeletal adaptations to concurrent HIIT and resistance training are highly context-dependent. Variability in outcomes across studies appears to be shaped by interacting factors, including participant characteristics, training status, intervention duration, and the relative emphasis placed on resistance loading versus interval intensity. Accordingly, the following discussion focuses on synthesizing these patterns to explain why certain adaptations are consistently observed under specific conditions, while others remain variable or attenuated.

### 4.1. Strength Adaptations

Between the evidence under study, there is a persistent improvement of maximal strength after concurrent HIIT and resistance training that would suggest that neural mechanisms are indeed the primary motivators for adaptation. In older and middle-aged individuals, modest increases in one repetition maximum strength and isometric force [23,24,30,31] are consistent with our finding that early strength increases in aged muscle are strongly supported by increased motor unit recruitment, better rate coding, and improved synchronization of motor unit firing [44,45]. The maintenance of strength gains with some or no hypertrophy, however, indicates that neural processes offer much of the early adaptation reserve in this group.

In recreationally active and athletically trained cohorts, maximal force and sport-specific strength were maintained or improved even when HIIT imposed substantial additional running or sprint work [27,28,29]. This pattern suggests that, when resistance loading is clearly prioritized, neural drive to prime movers can be preserved under mixed metabolic stress and that musculotendinous stiffness is maintained at levels sufficient to transfer force effectively during multi-joint tasks [44,45].

Although it is the case that there are structural processes contributing to strength adaptation, the prevalence seems high in younger adults. This hypertrophy-oriented process, as found in the resistance training literature [46], may facilitate the association between some concurrent protocols and modest improvements in lean tissue or fiber level indices in recreationally active adults [39]. By contrast, shorter or metabolically oriented interventions [26] had less exposure to heavy loading, and thus, these morphological effects may have been insufficient. Such an interaction between the neural and structural pathways agrees with models in which neural adjustments dominate early progression while hypertrophic remodeling consolidates strength over longer time frames.

Trainability molds the expression of these mechanisms. Two to three weekly resistance sessions and concise HIIT bouts in protocols may have maintained the mechanical loads that were required to promote neural and structural development. This configuration is in line with models focusing on the role of training frequency and weekly loading for the development of strength when combined [47].

In this mechanistic framework, concern about interference can be read in a narrower sense. Synthesis of literature from concurrent training studies has shown that molecular-level interference (e.g., mTOR-related signaling inhibition) is most likely induced when endurance volume is high, sessions are poorly sequenced, or recovery is poor [48,49]. Meta-analytic findings indicate that modality, intensity, and weekly distribution moderate this risk [50]. In contrast, the protocols examined in the present review adopted predominantly time-efficient HIIT and progressive resistance loading techniques, conditions in which neural and hypertrophic pathways may have remained intact.

In summary, the strength-related results lend an element of a mechanistic model suggesting concurrent HIIT and resistance training can potentially maintain or enhance maximal force level through neural adaptations with some added structural reinforcement when loading is sufficient, duration is adequate, and interference is attenuated by well-controlled endurance volume and recovery.

### 4.2. Power and Explosive Performance Adaptations

The maintenance or improvement of power-related outcomes under concurrent HIIT and resistance training indicates that rapid force production mechanisms can remain highly responsive even when additional interval work is imposed. At the neuromuscular level, explosive performance depends on the rate of force development, which reflects the speed of motor unit recruitment, discharge frequency, and intramuscular coordination [51]. Resistance and power training are known to increase efferent neural drive and reduce inhibitory input at the spinal level, thereby supporting faster rises in force and more efficient use of the stretch–shortening cycle [52].

Age-related declines in muscle power are associated with fewer type II motor units recruited and a lower peak rate of force development, changes that are significantly associated with functional impairments in older individuals [53]. The retention or advancement of jump and rapid force tasks seen in older and middle-aged individuals [23,30,31,54] is likely to indicate restoration of motor unit recruitment capacity and better coordination of agonist and synergist muscles, even when hypertrophic responses are small.

Rate of force development reviews show that high-intent, explosive contractions across a range of loads are especially effective in the development of muscular power, particularly when athletes or older adults are involved [51]. Models of programming related to power-oriented training stress the dual use of muscular training focused on strength and velocity, emphasizing a combination of force-training activities in high volume, ballistic motion, and plyometrics while balancing total volume and fatigue [55,56]. In this context, simultaneous protocols as described in the current review may be considered hybrids: HIIT sessions introduce neuromuscular and metabolic stress on the velocity end of the force–velocity spectrum, while resistance sessions sustain or enhance maximal force capacity.

Mechanistically, the most favorable power results seem to indicate that short, glycolytic HIIT does not substantially influence the neural adaptations involved in explosive performance as long as the strength training retains a significant power component and the fatigue is moderated. Instead, concurrent exposure to both high-force and high-velocity stimuli may broaden the spectrum of functional adaptations across the force–velocity curve.

### 4.3. Neuromuscular Activity (EMG) Adaptations

The limited but informative EMG data provide further insight into how neural mechanisms adapt under concurrent HIIT and resistance training. In older and middle-aged adults, unchanged or slightly reduced EMG amplitudes during submaximal tasks, combined with better task performance [23,24,30,31], are consistent with an efficiency-oriented adaptation profile. Established models of early strength and power adaptation emphasize improvements in motor unit recruitment, rate coding, and coordination rather than large changes in muscle size [44,45,51,52]. Producing the same or greater external work with lower relative EMG activation fits these models and suggests improved matching between neural drive and mechanical demand.

In recreational and sport populations, EMG indices were task dependent. At high-intent resistance exercise, with moderate-volume HIIT, activation during explosive or high-velocity movements was preserved or modestly increased [26,27,28,29,39,40]. These findings are consistent with evidence that repeated exposure to rapid force production promotes neural adaptations that support rate of force development, including faster recruitment of high-threshold motor units and higher discharge rates [51,52,55,56].

Several treatments, to the contrary, were associated with clear improvements in strength and power with little change in EMG amplitude. This disconnect is not surprising given the sensitivity of surface EMG to methodological parameters, including electrode placement, subcutaneous tissue thickness, contraction mode, and signal normalization [57]. This approach highlights the limitations of surface EMG as a single marker of neural drive and implies that additional, more extensive neuromuscular analyses are required to characterize concurrent training adaptations completely.

Collectively, the EMG-related results provide the basis for a mechanistic interpretation of concurrent HIIT/resistance training results that will induce enhanced neuromuscular efficiency in older adults and retain or strengthen task-specific activation in younger and athletic cohorts in explosive actions.

Taken together, these findings suggest that the preservation or enhancement of musculoskeletal performance under concurrent training is not determined by the presence of HIIT per se, but by how interval-based stimuli are integrated with resistance loading. Studies that maintained sufficient resistance training volume, intensity, and movement specificity tended to report more favorable strength and power outcomes, whereas protocols with a predominant metabolic emphasis showed more variable adaptations. This pattern underscores the importance of training configuration and supports a conditional rather than absolute interpretation of concurrent training effects.

### 4.4. Muscle Morphology and Architecture Adaptations

Mechanistic interpretation of morphological findings indicates that structural remodeling plays a supportive, rather than primary, role in the adaptations to concurrent HIIT and resistance training. In older and middle-aged adults, modest changes in muscle cross-sectional area, thickness, or architecture [23,24,30,31] are consistent with broader evidence that anabolic responsiveness is blunted with age and that improvements in function often outpace changes in muscle size [44,45,46]. Under these conditions, neural and qualitative changes in muscle tissue may account for a larger proportion of the performance gains.

In younger and/or physically tested subjects, more definite evidence of structural adaptation could be identified when the resistance loading was sufficiently progressive, and HIIT volume did not dominate the weekly workload [26,27,28,29,39,40]. The added muscle thickness, fiber-specific hypertrophy, and shift in form-shifting architecture imply the possible stimulation of hypertrophic pathways by the combination of a high degree of intent contractions and intermittent interval work upon sufficient recovery [46,58].

Architectural-specific adaptations exhibit characteristic mechanistic patterns. Increasing pennation angle and muscle thickness increases the available physiological cross-sectional area for force production, which in turn facilitates sustainable development of strength. In contrast, preservation or elongation of fascicle length tends to be more sensitive to rapid force transmission and sprinting ability [58,59]. Controlled training studies suggest that high-intent resistance exercise increases the pennation angle, and repeated stretch–shortening and abrupt changes in contraction modes, such as in HIIT, may have effects on the adaptability of fascicle length [52,55,56,59].

The low degree of architectural remodeling shown across trials in older and middle-aged adults is consistent with information that age-related losses in muscle function are only partly accounted for by reductions in muscle size and also reflect changes in muscle quality and neural drive [60]. The co-presence of stable morphology with superior function implies that morphological remodeling in this group is a more gradual and supplementary aspect of the overall adaptive profile.

These observations provide evidence that when conditioning individuals with simultaneous HIIT and resistance training (which can, when sufficiently loaded and prolonged, elicit significant structural and architectural changes, particularly among younger, trained subjects and among older adults), neural and qualitative adaptations are the primary sources of change.

### 4.5. Tendon Adaptations

Mechanistically, tendon adaptations constitute an important but largely inferential component of the concurrent training response. In the trials screened here, no direct imaging or mechanical evaluation of tendon stiffness, cross-sectional area, or viscoelastic properties was performed. Nevertheless, improvements in tasks that heavily depend on the muscle–tendon unit, such as rapid sit-to-stand transitions, jumping, and sprinting [23,24,28,29,31,32,33,34,35,38,39], suggest that tendon behavior likely contributed to the observed performance changes.

There is evidence of increased stiffness and cross-sectional area in tendons with repeated high loading and stretch–shortening exercise studies outside the current inclusion set [4,5,6]. Changes in the stiffness of the tendon will increase the speed and efficiency of force transmission and can enhance explosive performance even when hypertrophic changes in muscle are modest [58,59]. Age-associated changes in collagen turnover, vascularity, and matrix organization also seem to slow tendon remodeling in older adults [60] and might influence the rate of adaptation over standard intervention durations.

In the setting of concurrent HIIT and resistance exercise, tendons in the younger and trained population may have undergone structural or mechanical changes, as may be seen in the case of isolated resistance and plyometrics, particularly in those programs that integrate heavy lifting and high-velocity movements. For older subjects, some of the most important influences on tendon response are possibly stabilization of the muscle–tendon unit and improved efficiency of force transfer, as opposed to structural modification [61,62].

On the whole, the available mechanistic evidence indicates that tendon adaptation is likely occurring during concurrent training in parallel with neural and muscular changes, but the need for direct measurements is apparent and remains an area for further study.

### 4.6. Interference Considerations

Interference is still the central theoretical issue related to high-intensity interval and resistance training. Interference is commonly attributed to competition between endurance-related and hypertrophy-related signaling mechanisms, especially when endurance loading is prolonged or voluminous. Prolonged endurance exercise can suppress mTOR signaling, elevate AMPK activation, and shift intracellular priorities toward oxidative remodeling rather than protein accretion [48,49,63].

The recent review, however, indicates that the nature of these molecular interactions may be stimulus-dependent rather than definitive. Under conditions of short, intermittent, and primarily glycolytic endurance efforts, signaling pathways of strength and hypertrophy seem to be largely preserved, particularly with resistance exercise given sufficient external load. Inclusion of HIIT protocols tended to emphasize short, near-maximal yet time-efficient work intervals; the execution of short work intervals or a sufficiently broad break between resistance and strength trials; and the model’s emphasis on the sequence of sessions, training density, and recovery time windows [49,63].

Meta-analytic work on concurrent training has also shown that interference effects are most potent when endurance volume is high, and recovery is limited, while short training with high intensities, on the other hand, exerts minimal negative influence on strength adaptation or muscle hypertrophy [50]. Improvements in strength, power, and neuromuscular efficiency for older, recreationally active, and athletic cohorts are matched with stable or improved morphological indices, which are consistent with the current analysis.

Mechanistically, this interference sensitivity is likely to differ depending on the domain. Strength development seems more resistant to perturbation when mechanical loading remains the primary stimulus, while hypertrophy and tendon remodeling may be more sensitive to cumulative endurance volume and fatigue. Training age, recovery capacity, and pre-existing cardiorespiratory fitness are likely to further influence this balance among individual factors.

Combined, the mechanistic and performance evidence suggest an interpretation in which the available evidence does not consistently demonstrate substantial interference as an inherent consequence of concurrent HIIT and resistance training under the conditions examined, but rather a controllable design variable shaped by volume, intensity, sequencing, and recovery.

The substantial heterogeneity observed across studies has important implications for both the interpretation and clinical applicability of the findings. Differences in participant age, training status, and baseline neuromuscular capacity are likely to influence the magnitude and expression of adaptations to concurrent HIIT and resistance training. Similarly, variation in intervention duration, resistance training volume and intensity, session sequencing, and HIIT structure may contribute to divergent outcomes, particularly for strength and power-related measures.

From an applied perspective, this heterogeneity indicates that concurrent training effects should be interpreted relative to specific population profiles and program designs rather than as uniform responses. In clinical and community settings, protocols emphasizing adequate resistance loading and recovery may be more relevant for preserving musculoskeletal function, whereas in athletic contexts, careful balancing of interval intensity and resistance training demands is likely critical for optimizing performance-related outcomes. Taken together, heterogeneity across studies underscores the need for context-specific interpretation when translating evidence into practice.

The interpretation of the present findings should also consider the risk-of-bias profile of the studies included (Table 1). Although overall methodological quality was acceptable, several trials exhibited high or unclear risk of bias in key domains, including allocation concealment, blinding of outcome assessment, and adherence monitoring. While no formal weighting procedure was applied during synthesis, methodological quality informed the level of confidence assigned to specific outcomes. Findings derived primarily from studies with an elevated risk of bias were interpreted more cautiously, particularly for outcomes reliant on indirect or task-dependent measures, such as electromyography-derived indices or functional performance tests. Consequently, the synthesis emphasizes outcome-specific confidence rather than uniform interpretation across all domains, reinforcing the importance of integrating quality appraisal into the interpretative framework rather than treating it as a purely procedural step.

From a clinical and practical perspective, the findings of this review suggest that concurrent HIIT and resistance training may be most beneficial when specific programming conditions are met. Across studies, favorable musculoskeletal outcomes were more consistently observed when resistance training quality, load progression, and movement specificity were preserved, and when HIIT was implemented in short, time-efficient formats with adequate recovery. These conditions appear to mitigate potential interference effects while maintaining neuromuscular and performance-related adaptations.

Population-specific considerations are also warranted. In older and middle-aged adults, concurrent training may be particularly appropriate for supporting functional performance and neuromuscular efficiency, provided that recovery capacity and safety are prioritized. In recreationally active and athletic populations, concurrent HIIT and resistance training may offer a time-efficient strategy to maintain or enhance strength and power, especially when endurance volume is carefully controlled. Conversely, protocols characterized by excessive endurance volume or insufficient recovery may be less suitable for individuals with limited training tolerance or elevated fatigue risk.

Accordingly, clinicians and practitioners should interpret the benefits of concurrent training in relation to individual characteristics, training history, and program design rather than assuming uniform responses across populations. This context-dependent perspective may help optimize the practical application of concurrent HIIT and resistance training while aligning expectations with the strength of the available evidence.

### 4.7. Integrated Interpretation of Musculoskeletal Adaptations

The neural, structural, and tendon mechanistic integration yields a comprehensive account of the general adaptive profile induced by concurrent HIIT and resistance training. Neuromuscular adaptations are associated with the earliest and the strongest overall outcomes across adult populations. Reduced EMG amplitudes during sub-maximal tasks, together with improved rapid force production, may reflect enhanced neuromuscular efficiency, altered motor unit recruitment strategies, or task-specific coordination adaptations. However, given the inherent limitations of surface EMG, these findings should not be interpreted as direct evidence of specific neural mechanisms but rather as indicative of integrated neuromuscular adaptation [64].

Structural and architectural changes, although slower and more modest in some cohorts, provide additional support. In younger and recreationally active adults, small increases in muscle thickness and pennation angle when resistance loading is adequate are consistent with maintained hypertrophic potential despite the presence of HIIT [59]. In older and middle-aged adults, the combination of maintained or slightly improved muscle size with more pronounced gains in functional performance suggests that improvements in muscle quality, neural drive, and possibly tendon behavior may account for a larger share of the adaptation [60].

Several tendon-related mechanisms may plausibly contribute to the stability and functional behavior of the muscle–tendon unit during rapid movements. However, in the absence of direct tendon imaging or mechanical measurements in the included trials, such interpretations should be considered indirect and hypothesis-generating rather than definitive. In this context, prior mechanistic literature suggests that changes in tendon stiffness may influence elastic energy storage and release during high-velocity tasks [65], which may partially help contextualize the observed dissociation between modest hypertrophic responses and preserved or improved explosive performance in concurrent training studies. Accordingly, improvements in functional performance should be interpreted as reflecting integrated neuromuscular–muscle–tendon behavior, rather than as direct evidence of tendon remodeling in the absence of imaging or mechanical assessments. As such, tendon adaptations in the context of concurrent HIIT and resistance training should be regarded as an evidence gap in the current literature, rather than as an inferred outcome based on functional performance measures.

The converging stream of evidence also suggests that interference effects were minimal in the current review. Time-efficient HIIT formats do not seem to exert the sustained metabolic stress or fiber-type-specific signaling competition most associated with the reduction in resistance training adaptations. Neural pathways are resilient to short interval bouts, hypertrophic signaling can be preserved under sufficient loads, and tendon behavior is unlikely to deteriorate under balanced loading [66]. Taken together, these findings are consistent with a mechanistic model in which concurrent HIIT and resistance exercise may improve musculoskeletal function through partially overlapping and non-competing mechanisms.

Altogether, the composite interpretation supports the view that simultaneous training can be efficacious for enhancing musculoskeletal performance when load progression, recovery considerations, and the integration of endurance and resistance components are appropriately managed.

Future research in this field should move beyond generalized comparisons of concurrent training modalities and adopt more targeted designs that account for population-specific characteristics and training objectives. Studies in older adults should prioritize functional and neuromuscular outcomes alongside long-term adherence and safety, whereas investigations in recreationally active and athletic populations should more clearly define the balance between resistance loading and interval intensity to optimize strength and power adaptations.

In addition, greater standardization and transparency in reporting training variables, including session sequencing, resistance training volume and intensity, and HIIT structure, would facilitate cross-study comparison and synthesis. The inclusion of direct assessments of musculoskeletal structure and function, such as imaging-based measures of muscle and tendon properties, as well as longer intervention durations, may further clarify adaptation mechanisms and time-dependent responses. Collectively, these directions may help refine evidence-based recommendations for concurrent HIIT and resistance training across diverse adult populations.

To further enhance interpretability and reproducibility, future studies should more explicitly define key training variables within concurrent HIIT and resistance training interventions. With respect to training frequency, investigations should systematically compare two versus three weekly resistance training sessions when combined with HIIT, particularly in populations with limited recovery capacity. Session sequencing also warrants targeted examination, as separating HIIT and resistance training by sufficient recovery intervals or manipulating exercise order within the same session may differentially influence neuromuscular and hypertrophic adaptations.

Resistance training intensity and loading progression should be reported with greater precision, including relative load (%1RM), movement velocity intent, and proximity to volitional fatigue, as these factors appear critical for preserving strength and power adaptations under concurrent conditions. In addition, future trials should clearly document HIIT structure, including interval duration, intensity prescription, and work-to-rest ratios, to facilitate mechanistic comparison across studies. Collectively, greater standardization and transparent reporting of these training variables may enable more robust synthesis and support the development of evidence-based recommendations for concurrent HIIT and resistance training across diverse adult populations.

### 4.8. Limitations

This review has a number of limitations that affect the strength and scope of the mechanistic conclusions. Despite the comprehensive search strategy employed, the evidence for concurrent HIIT and resistance training remains uneven across populations, as most available studies involve small samples drawn primarily from athletic and older adult cohorts. This limits the ability to draw firm conclusions about the mechanisms involved and their applicability to broader clinical or community cohorts. The heterogeneity of training duration, exercise choice, session sequencing, and weekly workload also hampers the ability to isolate specific programming variables or to develop precise dose–response relationships.

Overall methodological quality was acceptable but uneven. Blinding was infrequently possible, adherence monitoring varied across studies, and neuromuscular outcomes such as EMG and tendon measurements were inconsistently reported. Moreover, the limited number of studies addressing muscle architecture and tendon behavior constrains mechanistic understanding in both domains. In addition, the lack of long-term follow-up precludes definitive conclusions regarding the durability of neural, structural, or tendon adaptations during concurrent training and whether divergence from resistance-only approaches emerges over time.

An additional consideration relates to the assessment of training adherence across included studies. While adherence rates were generally reported as high, the methods used to assess compliance varied between trials. In many studies, adherence was based on supervised attendance records or session completion logs, whereas others relied partly on self-reported training participation. This heterogeneity in adherence assessment may influence the precision and comparability of reported compliance rates and should be considered when interpreting the feasibility and tolerability of concurrent HIIT and resistance training interventions.

Notwithstanding these caveats, the available evidence provides a reasonably consistent mechanistic characterization of how concurrent HIIT and resistance training influence strength, power, and neuromuscular function. It also highlights several priorities for future research, including more standardized reporting of neuromuscular and tendon outcomes, clearer characterization of training structure, and longer-term interventions capable of tracking adaptation trajectories across multiple physiological domains.

A further limitation of the present review relates to the reliance on narrative synthesis. Although this approach was necessary given the substantial heterogeneity across studies in participant characteristics, training protocols, intervention duration, and outcome assessments, it precludes quantitative pooling of effect sizes and formal estimation of between-study variance. Consequently, the conclusions drawn from this review should be interpreted as descriptive and pattern-based rather than as definitive quantitative estimates. This limitation may reduce the generalizability of the findings and underscores the need for more standardized study designs to enable future meta-analytical synthesis.

In addition, the absence of prospective protocol registration represents a methodological limitation of the present review. Although this may increase susceptibility to selective reporting bias, this risk was mitigated by predefined eligibility criteria and transparent reporting in accordance with PRISMA 2020 recommendations.

Therefore, the absence of consistent interference findings should not be interpreted as definitive evidence of equivalence between concurrent and resistance-only training.

## 5. Practical Implications

The results of this review suggest a number of tangible principles for concurrent incorporation of HIIT and resistance training in both musculoskeletal health and performance care programs. It is a key challenge to maintain the quality of resistance loading. In older and middle-aged consumers, those involved in recreation and those who are athletes or tactical participants, programs that allowed two to three weekly resistance training sessions of clear increases in intensity were adequate to promote maximal strength development, even when HIIT was introduced to the same routine. In application, this entails achieving the planned load- and volume-level progression on the vital lifts and avoiding large, unplanned reductions in mechanical tension when an interval session occurs, as is intended.

Power and explosive performance are goals; as a result, programs must ensure that resistance classes have a unique emphasis on high velocity. The trials we have reviewed here suggest that concurrent formats are the most effective for explosive benefits when fast concentric lifts, ballistic movements, and low- to moderate-load exercises are performed with maximal intent. In older and middle-aged adults, including functional power activities (e.g., rapid sit-to-stand transitions and step-ups) has the potential to facilitate the transfer of neuromuscular adaptations into daily practice and may result in greater functional capacity and reduced fall risk [66]. In such groups, the focus is less on optimizing peak power for sport and more on ensuring adequate rapid force generation in everyday mobility trials.

Time-efficient HIIT formats allow targeting endurance and metabolic goals without imposing the high volumes typically associated with interference. When spaced appropriately within the weekly schedule, short interval protocols based on near maximal, intermittent efforts appear compatible with strength and power development. Where time is a limiting factor, such as in-season athletic contexts or local community programs, short HIIT sessions may be placed on non-lifting days or separated from heavy resistance work by several hours in order to limit cumulative fatigue. To athletes and physically active adults alike, this allows pre-season blocks to emphasize gains in repeated sprint ability alongside strength, while in-season periods focus more on maintaining aerobic fitness and neuromuscular performance with minimal disruption to power training.

For elderly people or those looking for primarily health-focused progress, strength training in combination with time-saving HIIT offers a balanced weekly structure. It can improve strength, functional ability, and cardiometabolic health while also reducing the training duration without putting in a lot of effort at work. Monitoring of recovery and individual responses is essential across all populations. Basic parameters such as session rating of perceived effort, self-reported fatigue, and efficiency in dominant strength and power assessments may inform small adaptation strategies to the distribution of HIIT and resistance practice over time. If there is evidence of an overabundance of fatigue or stagnation in strength and power levels, volume may need to be reduced, or recovery between techniques increased, in order to conserve positive adaptation.

All considered, the congruence of HIIT and resistance training seems to not only be feasible with musculoskeletal fitness targets but might also serve as an adaptable and time-effective means in different adult populations when loading, sequencing, and recovery are deliberately performed.

## 6. Conclusions

In summary, the available evidence indicates that concurrent high-intensity interval training combined with resistance training can improve or maintain musculoskeletal performance across diverse adult populations. However, these adaptations are not uniform and should be interpreted in light of substantial heterogeneity across studies. Variability in participant characteristics, training status, intervention duration, resistance loading schemes, and HIIT configurations, as well as differences in outcome measures, limits the generalizability of findings and precludes simple, universal conclusions.

Despite these limitations, consistent patterns emerge suggesting that musculoskeletal outcomes are largely preserved when resistance training quality, load progression, and recovery are appropriately maintained within concurrent training programs. Neuromuscular adaptations appear to be particularly robust across populations, whereas structural and tendon-related adaptations remain less consistently documented and warrant cautious interpretation.

Future research should prioritize population-specific study designs, greater standardization and transparency in reporting training variables, and the inclusion of direct assessments of musculoskeletal structure and function. Longer intervention durations and mechanistically informed protocols may further clarify how concurrent HIIT and resistance training can be optimally integrated to support musculoskeletal health and performance.

## Figures and Tables

**Table 1:** Risk of bias summary for included trials.

Study (Ref.)	Design	Overall Risk of Bias	Main Concerns
García Pinillos et al., 2019 [23]	Controlled training trial in healthy older adults	Some concerns	Randomization and allocation procedures not clearly described; no blinding; small sample.
Wadsworth et al., 2022 [24]	Single-group pre–post concurrent program	High	No control group; no randomization; self-selection and maturation effects cannot be excluded.
Panissa et al., 2018 [25]	Randomized parallel-group training study	Some concerns	Allocation concealment not reported; no blinding; modest sample size.
Benítez-Flores et al., 2019 [26]	Randomized short-term parallel trial	Some concerns	Very short duration; limited information on allocation concealment and blinding.
Pugh et al., 2015 [27]	Acute randomized crossover trial	Some concerns	Small sample; no blinding; short follow-up limited to acute responses.
Campos Vázquez et al., 2015 [28]	Randomized preseason training study	Some concerns	Team-sport setting, potential contamination between groups; allocation procedures not fully detailed.
Botonis et al., 2016 [29]	Non-randomized in-season controlled study	High	Group allocation not randomized; small sample; potential confounding from team practice and schedule.
Wong et al., 2010 [30]	Randomized preseason team-sport trial	Some concerns	Limited reporting on allocation concealment and blinding; potential performance bias in team environment.
Müller et al., 2021 [31]	Randomized controlled trial in older men	Some concerns	Participant and trainer blinding are not feasible; some outcomes based on performance tests are susceptible to expectation effects.
Vlietstra et al., 2023 [32]	Randomized controlled trial in middle-aged adults	Some concerns	No blinding of participants; adherence threshold may exclude less-compliant individuals and introduce selection bias.
Thomakos et al., 2023 [33]	Randomized preseason soccer trial	Some concerns	Team context and shared training environment may lead to contamination; blinding not reported.
Thomakos et al., 2024 [34]	Randomized in-season soccer trial	Some concerns	Player blinding not feasible; differential match exposure may influence outcomes.
Robineau et al., 2017 [35]	Randomized parallel-group rugby sevens trial	Some concerns	Attrition across groups; no blinding; training outside the study protocol not fully controlled.
Leuchtmann et al., 2020 [36]	Randomized controlled trial in older men	Some concerns	Blinding of participants not possible; some outcomes based on sub-samples (biopsies).
Kazior et al., 2016 [37]	Controlled training study in young men	Some concerns	Randomization and allocation procedures incompletely reported; small sample; no blinding.
Spiliopoulou et al., 2021 [38]	Randomized concurrent power + HIIT trial	Some concerns	No blinding; modest sample size; some imaging outcomes based on small subgroups.
Sterczala et al., 2023 [39]	Randomized concurrent training trial in military-aged adults	Some concerns	No blinding; complex multi-component program; occupational tests partly field-based and operator-dependent.
Sterczala et al., 2024 [40]	Randomized high-intensity, low-volume concurrent trial	Some concerns	Short-to-moderate duration; limited description of allocation concealment and blinding; mechanistic outcomes based on biopsy sub-samples.

**Table 2:** Characteristics of included studies.

Study (Ref.)	Population	Design and Duration	HIIT/Sprint Component	Resistance/Power Component	Comparator Condition(s)	Primary Musculoskeletal Outcomes and Main Findings
Wadsworth et al., 2022 [24]	Inactive aging women, ~40–64 years; apparently healthy but low active	Randomized, 10-week concurrent training program combining sprint intervals and undulating resistance training; ~3 sessions/week	Treadmill sprint-interval training with very short, high-intensity work bouts (≈40 s close to ≥90–95% HRmax) interspersed with brief passive recovery; performed 2 times/week as part of the concurrent program	Non-linear (undulating) resistance training across major muscle groups, progressing over 30 sessions; included both moderate and higher load phases to target strength and muscular endurance	Single concurrent-training group (no pure RT control); outcomes evaluated pre–post within group	1RM strength, body composition, and functional performance tests. The 10-week program improved lower-body 1RM and functional outcomes, with modest changes in body composition. Strength gains occurred despite the inclusion of frequent high-intensity sprints, suggesting compatibility of sprint-interval work with resistance training in middle-aged women.
Panissa et al., 2018 [25]	Young men, recreationally trained; free of major disease or injury	Randomized, 8-week training study; 3 sessions/week of either concurrent training or strength-only	High-intensity intermittent running or cycling intervals performed shortly before or after the strength portion in the concurrent group; brief repeated work bouts at vigorous to near-maximal intensity with controlled recovery	Multi-joint and single-joint resistance exercises for upper and lower body, performed with progressive loading (moderate to high loads across sets; volume recorded to calculate total volume load)	Strength-only group performing the same resistance training without HIIT; concurrent group combining HIIT plus the same strength exercises	Primary outcome was maximal dynamic strength (1RM in key lifts) and total resistance training volume. Both groups improved 1RM strength, but strength-only tended to accumulate higher volume load. The addition of HIIT did not abolish strength gains but slightly constrained volume progression, illustrating a manageable but detectable concurrent effect on training load.
Benítez-Flores et al., 2019 [26]	Young men, recreationally active; no major health issues	Randomized, 2-week intervention with 6 sessions; short-term concurrent sprint plus resistance training vs. single-mode training	Very short “all-out” sprints (≈5 s) performed repeatedly on a cycle ergometer or treadmill, with brief recovery between efforts; sprints integrated alongside resistance work in the combined protocol	Lower- and upper-body resistance exercises (multi-joint dominant), performed with moderate to high loads; the concurrent group performed resistance plus sprints within the same sessions	Groups included: sprint-plus-resistance vs. resistance-only vs. sprint-only (and/or control, depending on the exact design)	Neuromuscular performance (jump tests, short sprint performance), aerobic/anaerobic markers, and heart rate variability. Even over 2 weeks, combined sprint and resistance training produced meaningful improvements in aerobic and anaerobic performance without compromising strength-related outcomes. Short all-out efforts appeared to be well tolerated when paired with moderate-volume resistance training.
Pugh et al., 2015 [27]	Young, untrained men; healthy	Acute randomized crossover trial; one session of concurrent resistance plus HIIT, with muscle biopsies pre- and post-	High-intensity interval cycling performed either before or after a bout of resistance exercise; repeated work bouts at vigorous intensity with fixed recovery intervals	Single resistance-training bout emphasizing lower body multi-joint work at moderate to heavy loads; designed to stimulate strength/hypertrophy pathways	Conditions compared sequence (RT → HIIT vs. HIIT → RT) and/or HIIT-only or RT-only	Primary endpoints were acute molecular and signaling responses in skeletal muscle (e.g., AMPK, mTOR-related pathways) and markers of substrate metabolism. The study showed that a single concurrent session can activate both endurance- and strength-related signaling, with sequence influencing some molecular responses. Provides mechanistic context for chronic adaptations observed in longer trials.
Campos Vázquez et al., 2015 [28]	Young male soccer players; competitive youth athletes	Randomized, 7-week preseason study; 3x/week training combining repeated sprints and strength vs. alternative strength method	Field-based repeated sprint training with short high-intensity runs (e.g., 30 m sprints) interspersed with brief recovery; integrated into soccer-specific conditioning	Two different strength-training approaches (e.g., traditional vs. velocity-oriented) over lower-body multi-joint exercises; progressive loading across the 7 weeks	Two concurrent training groups (same sprint training, different strength method); no pure endurance-only group	Outcomes included 1RM or near-maximal strength, jump performance, repeated-sprint ability, and field-test measures. Both concurrent protocols improved strength and sprint-related performance, with some differences between the strength methods. The addition of high-intensity repeated sprints did not impair strength gains and supported soccer-specific performance.
Botonis et al., 2016 [29]	Elite male water polo players; high-level competitive athletes	In-season concurrent training study (~8 weeks), with players allocated to combined strength + interval endurance training vs. usual practice	Pool-based interval endurance training with repeated high-intensity swims at or above race pace, interspersed with structured recovery; frequency integrated with team practice	Dry-land strength training emphasizing multi-joint upper and lower body exercises, performed with moderate to heavy loads to improve maximal strength and power	Strength + interval endurance vs. more traditional team conditioning, with both groups continuing water polo practice	Main outcomes were maximal strength, swimming performance (e.g., repeated sprint swims, aerobic capacity), and water polo-specific test results. Players performing concurrent strength plus high-intensity interval swimming improved or maintained strength while enhancing water polo-specific endurance, indicating that well-planned concurrent work can support musculoskeletal performance in aquatic team sports.
Wong et al., 2010 [30]	Professional male soccer players; elite level	Preseason randomized study (~10 weeks) comparing concurrent strength + HIIT vs. control conditions within standard team training	High-intensity running intervals (e.g., repeated runs at close to maximal aerobic velocity or above), integrated 2–3 times per week during preseason	Structured muscular strength sessions targeting lower and upper body, using free weights and machines at moderate to high loads; focused on maximal and explosive strength relevant to soccer	Strength + HIIT group vs. group with more traditional conditioning (e.g., moderate-intensity running) or less systematic strength exposure	Outcomes included 1RM strength, jumping ability, sprint performance, and aerobic fitness tests. Concurrent strength + HIIT improved lower-body strength and power while also enhancing aerobic indices. The study showed that preseason integration of HIIT does not necessarily compromise strength development in professional soccer players when resistance training is adequately maintained.
Müller et al., 2021 [31]	Healthy older men (~65–80 years), insufficiently active but without major disability	Randomized controlled trial, 12 weeks; supervised 3x/week	High-intensity interval cycling performed at near-maximal intensities (e.g., short repeated intervals around 85–95% peak capacity) with controlled work–rest structure	Traditional strength-training group performed multi-joint and single-joint exercises with progressive heavy loading; the power group emphasized higher movement velocity at moderate loads; both combined with HIIT depending on allocation	HIIT + traditional strength vs. HIIT + power-oriented strength vs. (potentially) a more basic training condition; all in a structured laboratory setting	Primary outcomes were lower-extremity strength, muscle power, gait and chair-rise performance, and cardiorespiratory fitness. Both combined protocols improved functional capacity and muscular performance, with some added benefits of power-oriented work for rapid-force tasks. HIIT did not prevent strength or power gains in older men and appeared to support functional improvements.
Vlietstra et al., 2023 [32]	Middle-aged adults with low relative lean soft tissue mass; men and women	Randomized controlled trial, 12 weeks; 3 supervised sessions/week	High-intensity interval aerobic training (likely cycling or treadmill) performed with repeated high-intensity work intervals and matched recovery periods	Resistance training targeting major muscle groups with progressive loading, designed to increase lean mass and strength	Combined HIIT + RT vs. comparator (e.g., moderate-intensity continuous training, RT-only, or standard care depending on design)	Primary endpoints were lean soft tissue mass (via DXA), muscle strength, and physical function. Combined HIIT and resistance training increased lean mass, improved strength, and enhanced physical performance compared with baseline and, in some outcomes, relative to comparison conditions. The protocol effectively counteracted low lean mass without compromising strength gains.
Thomakos et al., 2023 [33]	Young male soccer players; competitive youth	Preseason randomized study (~7–8 weeks) comparing concurrent high-intensity and strength training formats	Field-based high-intensity running or sprint intervals performed 2–3 times/week, often in small-sided or shuttle formats mimicking match demands	Lower-body strength and power exercises (e.g., squats, jumps) implemented with progressive overload to target maximal strength and explosive performance	Combined strength + HIIT vs. alternative conditioning regimen or control approach within team training	Outcomes included countermovement jump, sprint times, aerobic performance, and sport-specific tests. Concurrent high-intensity and strength training enhanced muscle power and aerobic capacity, demonstrating that well-structured preseason concurrent work can simultaneously improve neuromuscular and endurance qualities in young soccer players.
Thomakos et al., 2024 [34]	Young male soccer players; same team context as [33] but in-season	In-season randomized study (~6–8 weeks) testing two brief HIIT formats, both combined with ongoing strength and soccer practice	Two short high-intensity interval formats differing in work:recovery structure or mode (e.g., long vs. short intervals), both implemented in addition to regular training	Strength training continued during the in-season period, focusing on maintenance of maximal strength and power through moderate volume, higher-intensity sets	Two concurrent HIIT conditions (e.g., format A vs. format B) with similar strength and technical training; no pure non-HIIT group	Main outcomes were aerobic performance, neuromuscular tests (jumps, sprints), and repeated-sprint ability. Both HIIT formats maintained or improved aerobic capacity without substantial loss of strength or power across the in-season, suggesting that short HIIT additions can be used flexibly without heavy interference with neuromuscular performance.
Robineau et al., 2017 [35]	Amateur male rugby sevens players (*n* ≈ 36)	Randomized, 8-week concurrent program; 3 groups: strength + short-interval HIIT, strength + sprint-interval training, and strength-only	Short-interval training (INT) used repeated high-intensity efforts of moderate duration, whereas sprint-interval training (SIT) consisted of very brief near-maximal sprints; both performed in addition to strength	Strength program common to all groups, using multi-joint lifts at moderate to high loads to develop maximal strength and power	INT (strength + short intervals), SIT (strength + sprints), and strength-only (CON)	Outcomes included maximal strength, power tests, V̇O_2_peak, maximal aerobic velocity, and rugby-specific repeated-sprint ability. All groups improved strength, while INT and SIT produced superior gains in aerobic measures and repeated-sprint performance. Strength was preserved despite additional high-intensity running, showing that concurrent configurations can be tailored in team sport settings.
Leuchtmann et al., 2020 [36]	Healthy older men; previously inactive or recreationally active	Randomized controlled trial, 12 weeks; initial HIIT phase followed by RT or continued HIIT	High-intensity interval cycling used as initial stimulus to improve cardiorespiratory fitness and muscle perfusion; intervals performed at high relative intensity with structured work–rest cycles	After initial HIIT, one group continued RT with heavy loads to preserve and augment HIIT-induced skeletal muscle capillarization; exercises targeted major lower-body muscle groups	Groups included HIIT followed by RT vs. alternative continuation (e.g., HIIT-only or different sequence)	Primary outcomes were skeletal muscle capillarization (histological measures), muscle function, and aerobic capacity. HIIT improved capillarization, and subsequent RT preserved these microvascular gains while also supporting strength improvements. Findings support the compatibility of sequential HIIT and heavy resistance training for microvascular and functional adaptations in older men.
Kazior et al., 2016 [37]	Healthy young men; recreationally active	Controlled training study (≈7–8 weeks), comparing strength training alone vs. strength plus endurance exercise	Endurance work consisted of cycling or running at moderate to high intensity, performed either concurrently with or in addition to strength workouts	Strength program focused on lower-body resistance training (e.g., leg press/extension) at moderate to high loads; same RT in both groups	Strength-only vs. concurrent strength + endurance group	Muscle biopsies assessed fiber cross-sectional area and signaling proteins (Akt, mTOR, etc.), along with strength performance. Endurance exercise enhanced some hypertrophic and molecular responses when added to strength training, with larger increases in fiber size and protein expression than strength-only in certain muscles. The study suggests that appropriately dosed endurance work need not blunt, and may even augment, morphological adaptations.
Spiliopoulou et al., 2021 [38]	Young men; recreationally trained	Randomized, 8-week concurrent program combining power training and HIIT cycling vs. control	High-intensity interval cycling sessions with repeated short bouts at vigorous to near-maximal intensity; added 2–3 times/week	Power-oriented resistance training for lower limbs (e.g., squats, jumps) performed at moderate loads with high movement velocity, designed to enhance rate of force development	Power + HIIT vs. strength/power training alone or habitual activity (depending on the design)	Outcomes included muscle morphology (e.g., quadriceps cross-sectional area via imaging), architecture indices, maximal strength, jump performance, and cycling power. Concurrent power training and HIIT produced improvements in muscle size and power output without clear evidence of interference, suggesting that explosive RT can coexist with high-intensity cycling in young men.
Sterczala et al., 2023 [39]	Recreationally active men and women; military-aged adults	12-week randomized study; concurrent resistance + interval training vs. comparison, focusing on occupational performance	High-intensity interval sessions (e.g., loaded or unloaded intervals) implemented alongside resistance training, targeting both aerobic capacity and task-specific tolerance	Comprehensive resistance program focused on whole-body strength and power relevant to military tasks; multi-joint lifts, progressive loading	Concurrent training group compared with alternative conditioning (e.g., traditional physical training)	Primary outcomes were maximal strength, power, V̇O_2_max, and military occupational task performance (e.g., loaded marches, lifts). Concurrent resistance and interval training improved occupational task performance and strength in both sexes, indicating that this configuration is effective for applied military settings without compromising musculoskeletal performance.
Sterczala et al., 2024 [40]	Recreationally active men and women	8–12-week trial (high-intensity, low-volume concurrent program)	Low-volume interval training at high intensity (brief, repeated efforts with relatively small total work time per session)	High-intensity, low-volume resistance training focusing on heavy multi-joint lifts, performed with limited total sets but high effort	Concurrent high-intensity, low-volume conditioning; often contrasted with baseline or standard training	Muscle biopsies and performance tests evaluated skeletal muscle adaptations (fiber size, strength, power, and molecular markers). The high-intensity, low-volume concurrent program elicited significant neuromuscular and structural adaptations in men and women, showing that relatively small amounts of carefully targeted concurrent work can meaningfully enhance musculoskeletal function without large training volumes.

**Table 3:** Summary of musculoskeletal outcomes by population group in concurrent HIIT plus resistance training trials.

Trials (Ref.)	Population Group	Strength (1RM/MVC)	Power/Explosive (CMJ, Sprint, RFD)	Neuromuscular Activity (EMG)	Morphology/Architecture (CSA, FL, PA)	Tendon (Stiffness/Strain)	Functional/Task Performance
[23,24,31,32,36]	Older adults (≥60 y)	↑ small–moderate in major lifts and isometric tests	↑ or ↔; gains mainly in chair-rise and gait speed; limited data on CMJ	↓ EMG at submax loads (efficiency ↑); ↑ activation capacity in some tests	↑ or ↔ muscle CSA; modest changes in fascicle length and pennation when reported	↔ to slight ↑ in stiffness; HIIT did not blunt RT-related tendon adaptations	↑ sit-to-stand, walking capacity, composite function scores; better maintenance of daily tasks
[25,26,27,37,38,40]	Middle-aged/recreational adults	↑ moderate–large when RT volume/progression maintained; ↔ when RT dose low or very short-term	↑ CMJ height, RFD, or sprint performance in most multi-week protocols; ↔ in very short (≤2 wk) interventions	Mixed: ↑ peak activation and rate of activation in high-intent tasks; some ↓ EMG at submax loads (efficiency)	↑ fiber CSA and lean mass in several trials; architecture changes (FL, PA) direction depends on power vs. strength emphasis	Limited direct tendon measures; no evidence of deterioration with concurrent formats	↑ composite fitness scores, work-related tasks, and muscular endurance; equivocal changes when programs are very short or under-dosed
[28,29,30,33,34,35,39]	Athletes/tactical populations	↑ or maintained maximal strength despite added HIIT; decrements rare when heavy RT preserved	↑ CMJ, sprint, repeated-sprint performance in most formats; isolated ↓ CMJ when HIIT volume is very dense or poorly sequenced	↑ EMG amplitude and rate of activation during explosive tasks in some trials; pattern often task-specific	Small ↑ in CSA and lean mass in preseason or off-season periods; in-season protocols mainly maintain morphology	Tendon outcomes rarely measured; no reports of impaired function; indirect indicators (stiffness-related performance) generally preserved	↑ sport-specific or occupational performance (Yo-Yo, RSA, rugby sevens tests, military tasks); maintenance of match fitness across congested periods

Note: ↑ = improvement or increase; ↓ = reduction; ↔ = no clear change or mixed findings.

**Table 4:** Training design features associated with favorable versus equivocal adaptations.

Domain	Favorable Patterns in Concurrent HIIT + RT	Equivocal/Less Favorable Patterns	Representative Trials (Ref.)
Maximal strength	2–3 RT sessions·wk^−1^ with progressive loading; HIIT 1–3 sessions·wk^−1^, mostly cycling or short running intervals; clear priority on multi-joint RT and lower-body strength; HIIT scheduled on separate days or after upper-body work	Very short interventions (≤2 wk); low RT volume or lack of progression; frequent lower-body HIIT immediately before heavy RT; high endurance load with limited recovery	Strength gains and maintenance in older and recreational adults [23,24,25,31,32,40]; team-sport and tactical populations maintaining or improving strength in-season [28,29,30,33,34,35,39]
Power/explosive performance	Inclusion of velocity-focused RT (light–moderate loads lifted with maximal intent); plyometric or jump-based elements; sprint- or RSA-type HIIT closely matching sport demands; HIIT performed after RT or on separate days	Programs with little or no power-oriented RT; very dense HIIT blocks with limited recovery; in-season periods where HIIT volume increases but RT intensity or frequency drops	CMJ and sprint improvements in athletes and active adults [25,28,29,30,33,35,38,39]; modest gains in older adults when explosive tasks were included [23,31,32]
Neuromuscular activation (EMG)	Emphasis on high-intent contractions (fast concentric phase); moderate session duration to avoid excessive fatigue; intervals structured to allow quality efforts across sets	Very fatiguing sessions with long total HIIT duration and minimal rest; low-load RT performed slowly without intent; very short interventions where neural changes may not fully develop	EMG efficiency gains and improved activation patterns in older and recreational adults [23,24,38,40]; sport-specific activation maintained or enhanced in athletes [28,29,33,39]
Muscle morphology/architecture	Multi-joint RT at moderate–high loads (≈70–85% 1RM); 2–3 RT sessions·wk^−1^; concurrent HIIT kept time-efficient (≤30 min) and not performed to exhaustion every session; interventions ≥8–12 wk	Protocols dominated by HIIT with minimal RT sets; very short (≤2 wk) programs; in-season phases where RT is de-emphasized; insufficient weekly volume for hypertrophy in trained athletes	CSA and lean mass gains in middle-aged and recreational populations [25,37,38,40]; preservation or small increases in older men when RT was maintained [31,36]; mixed architectural changes across age groups [23,37,38]
Tendon behavior	Progressive RT with sufficient load and tempo control; HIIT volume moderated so cumulative lower-limb loading is manageable; longer intervention duration (≥12 wk) when tendon change is a target	Short-term protocols where structural remodeling is unlikely; very high impact or downhill running without gradual progression; lack of any heavy slow resistance component	Maintenance of tendon-related function in older men undergoing concurrent HIIT + RT [31,36]; indirect stiffness-sensitive performance preserved in athletes [28,29,30,33,35]
Functional and task performance	Programs that integrate both strength and aerobic endpoints into periodization; HIIT tailored to functional tasks (e.g., loaded marches, change-of-direction); RT focused on key occupational or sport tasks (squats, deadlifts, pulls, pushes)	Mismatch between training content and target tasks; insufficient total training duration; focus on laboratory outcomes with limited transfer to real-world demands	Improvements in daily function in older adults [23,24,31,32]; better match performance in team-sport athletes [28,29,30,33,35]; enhanced occupational task performance in military personnel [39,40]

## Data Availability

No new data were created or analyzed in this study.

## Supplementary Materials

The following supporting information can be downloaded at: https://www.mdpi.com/article/10.3390/life16030381/s1, all search strategies, adherence and safety reporting, outcome domains and primary measures, exclusion lists, RoB 2 rationales, PRISMA 2020 checklist, full-text exclusions (PRISMA-compliant summary), and study-level log of full-text records excluded.

## Abbreviations

The following abbreviations are used in this manuscript:

1RM	One repetition maximum
CMJ	Countermovement jump
CON	Control group (strength-only or comparison condition)
CSA	Cross-sectional area
DXA	Dual-energy X-ray absorptiometry
EMG	Electromyography
FL	Fascicle length
HIIT	High-intensity interval training
HRmax	Maximal heart rate
INT	Short-interval training (interval training condition)
MVC	Maximal voluntary contraction
PA	Pennation angle
PICO	Population, Intervention, Comparison, Outcome
PRISMA	Preferred Reporting Items for Systematic Reviews and Meta-Analyses
RFD	Rate of force development
RSA	Repeated-sprint ability
RT	Resistance training
SIT	Sprint-interval training
V̇O_2_max	Maximal oxygen uptake
V̇O_2_peak	Peak oxygen uptake
