# Supplementary material for: Concurrent HIIT and Resistance Training for Musculoskeletal Function: A Systematic Review of Neuromuscular, Morphological, and Performance Adaptations
Source: Life (Basel). 2026 Feb 27;16(3):381. doi: 10.3390/life16030381 (PMC13028498; doi:10.3390/life16030381)
Supplement: Supplementary file 1 [file life-16-00381-s001.zip › MDPI-LIFE-SR-Table S1.pdf]

Table S1. Database-specific search strategies

Database	Platform	Date of last search	Fields	Search string (truncated example)	Limits
PubMed	NCBI	15 December 2025	Title/Abstract, MeSH	(“high intensity interval training”[tiab] OR HIIT[tiab] OR “sprint interval”[tiab]) AND (resistance[tiab] OR “strength training”[tiab] OR “weight training”[tiab]) AND (muscle[tiab] OR neuromuscular[tiab] OR musculoskeletal[tiab]) AND (adult[MeSH Terms] OR athlete*[tiab])	Humans; adults ≥ 18 years; English
Web of Science Core Collection	Clarivate	15 December 2025	Topic (Title, Abstract, Keywords)	TS = ((“high intensity interval” OR “high intensity intermittent” OR HIIT OR “sprint interval”) AND (resistance OR “strength training” OR “weight training”) AND (muscle OR neuromuscular OR musculoskeletal) AND (adult* OR athlete*))	Article; English
Scopus	Elsevier	15 December 2025	Title, Abstract, Keywords	TITLE-ABS-KEY((“high intensity interval” OR “high intensity intermittent” OR HIIT OR “sprint interval”) AND (resistance OR “strength training” OR “weight training”) AND (muscle OR neuromuscular OR musculoskeletal) AND (adult* OR athlete*))	Article; humans; English
PsycINFO	EBSCOhost	15 December 2025	Abstract, Subject Headings	AB((“high intensity interval” OR HIIT OR “sprint interval”) AND (resistance OR “strength training”) AND (muscle OR neuromuscular OR performance)) AND SU(exercise OR physical activity)	Peer-reviewed journal; adults; English
