# Supplementary material for: Concurrent HIIT and Resistance Training for Musculoskeletal Function: A Systematic Review of Neuromuscular, Morphological, and Performance Adaptations
Source: Life (Basel). 2026 Feb 27;16(3):381. doi: 10.3390/life16030381 (PMC13028498; doi:10.3390/life16030381)
Supplement: Supplementary file 1 [file life-16-00381-s001.zip › MDPI-LIFE-SR-Table S2.pdf]

Table S2. Full-text exclusions (PRISMA-compliant summary)

Primary exclusion reason	Operational definition/examples	n (full texts)	Notes / PRISMA 2020 items
Not concurrent HIIT+RT intervention	Full text describes exercise training but does not implement a concurrent high-intensity interval training plus resistance training protocol as defined in the eligibility criteria (for example, only HIIT, only resistance training, or other endurance or circuit programs without a combined HIIT+RT structure).	22	Excluded at full-text screening because the intervention did not match the target concurrent HIIT+RT exposure; contributes to PRISMA 2020 Item 16b.
No musculoskeletal outcomes	The study does not report prespecified musculoskeletal outcomes such as maximal strength, explosive performance, neuromuscular activation, muscle morphology or architecture, tendon-related measures, or functional performance tests.	14	Excluded at full-text screening because no eligible musculoskeletal endpoints were available for synthesis; contributes to PRISMA 2020 Item 16b.
Not controlled design	The study does not include a controlled comparison (for example, single-group pre–post design, case series, or uncontrolled feasibility trial), so it does not meet the predefined controlled-trial design criteria.	9	Excluded at full-text screening because there was no control or comparator condition; contributes to PRISMA 2020 Item 16b.
Wrong population	Participants fall outside the target healthy adult or athletic populations, for example, clinical cohorts, exclusively elderly or pediatric samples, or mixed samples where the eligible subpopulation cannot be isolated.	7	Excluded at full-text screening because the population did not match the predefined eligibility criteria; contributes to PRISMA 2020 Item 16b.
Insufficient methodological detail	Full text does not provide enough methodological information (for example, incomplete description of training protocol, sample characteristics, or outcome assessment) to determine eligibility or to interpret musculoskeletal adaptations.	5	Excluded at full-text screening because essential methodological details were missing; contributes to PRISMA 2020 Item 16b.

Total full-text records excluded: n = 57.

Note: In line with PRISMA 2020 Item 16b, full-text exclusions are summarized here according to standardized primary reasons. A study-level log with citation details and the specific exclusion reason for each report can be made available to editors and reviewers upon request.
