# Supplementary material for: Concurrent HIIT and Resistance Training for Musculoskeletal Function: A Systematic Review of Neuromuscular, Morphological, and Performance Adaptations
Source: Life (Basel). 2026 Feb 27;16(3):381. doi: 10.3390/life16030381 (PMC13028498; doi:10.3390/life16030381)
Supplement: Supplementary file 1 [file life-16-00381-s001.zip › MDPI-LIFE-SR-Table S2a.pdf]

**Table S2a. Study-level log of full-text records excluded at full-text screening**

| No. | First author, year | Article title | Journal/source | Population and setting | Brief description of intervention/design | Primary exclusion reason | DOI |
|---|---|---|---|---|---|---|---|
| 1 | Orlando, 2023 | Muscular Adaptations to Concurrent Resistance Training and High-Intensity Interval Training in Adults with Type 2 Diabetes: A Pilot Study | International Journal of Environmental Research and Public Health | Adults with type 2 diabetes; outpatient clinical setting | Pilot controlled study comparing concurrent HIIT+RT vs comparator; primary focus on diabetes-related outcomes. | Wrong population (clinical cohort) | https://doi.org/10.3390/ijerph20186746 |
| 2 | Pugh, 2018 | Satellite Cell Response to Concurrent Resistance Exercise and High-Intensity Interval Training in Sedentary, Overweight/Obese, Middle-Aged Individuals | European Journal of Applied Physiology | Sedentary overweight/obese middle-aged adults; laboratory setting | Acute mechanistic crossover; biopsy-based cellular outcomes; not a longitudinal training trial. | Acute mechanistic study (no multiweek adaptation outcomes) | https://doi.org/10.1007/s00421-017-3721-y |
| 3 | Pugh, 2015 | Acute Molecular Responses to Concurrent Resistance and High-Intensity Interval Exercise in Untrained Skeletal Muscle | Physiological Reports | Untrained adults; laboratory setting | Acute crossover comparing RT alone vs RT+HIIT; molecular signaling endpoints. | Acute mechanistic study (no longitudinal musculoskeletal outcomes) | https://doi.org/10.14814/phy2.12364 |
| 4 | Bettariga, 2024 | Effects of Resistance Training vs High-Intensity Interval Training on Body Composition, Muscle Strength, Cardiorespiratory Fitness, and Quality of Life in Survivors of Breast Cancer | Breast Cancer Research and Treatment | Breast cancer survivors; oncology exercise setting | RT-only vs HIIT-only arms; no concurrent HIIT+RT condition. | Not concurrent HIIT+RT intervention | https://doi.org/10.1007/s10549-024-07559-5 |
| 5 | Bettariga, 2025 | A Single Bout of Resistance or High-Intensity Interval Training Increases Anti-Cancer Myokines and Suppresses Cancer Cell Growth In Vitro in Survivors of Breast Cancer | Breast Cancer Research and Treatment | Breast cancer survivors; acute laboratory trial | Single-session RT vs HIIT; anti-cancer myokine / in vitro outcomes. | Wrong population and acute mechanistic | https://doi.org/10.1007/s10549-025-07772-w |
| 6 | Papadopoulos, 2021 | High-Intensity Interval Training or Resistance Training Versus Usual Care in Men with Prostate Cancer on Active | Applied Physiology, Nutrition, and Metabolism | Men with prostate cancer on active surveillance; clinic-based | Feasibility RCT with HIIT-only and RT-only arms; no concurrent HIIT+RT condition. | Not concurrent HIIT+RT intervention | https://doi.org/10.1139/apnm-2021-0365 |

| | | | | | | | |
|---|---|---|---|---|---|---|---|
| | | Surveillance: A 3-Arm Feasibility Randomized Controlled Trial | | | | | |
| 7 | Álvarez, 2024 | Six Weeks of a Concurrent Training Therapy Improves Endothelial Function and Arterial Stiffness in Hypertensive Adults with Minimum Non-Responders | Hipertensión y Riesgo Vascular | Adults with hypertension; clinical setting | Concurrent training emphasizing vascular endpoints (FMD, PWV). | Wrong population and outcomes not aligned | https://doi.org/10.1016/j.hipert.2024.07.001 |
| 8 | Meng, 2020 | Effects of Concurrent Aerobic and Resistance Exercise in Frail and Pre-Frail Older Adults: A Randomized Trial of Supervised Versus Home-Based Programs | Medicine (Baltimore) | Frail/pre-frail older adults | Concurrent aerobic+RT, not HIIT; frailty/functional outcomes. | Wrong population / not HIIT-based | https://doi.org/10.1097/MD.0000000000021187 |
| 9 | Baltasar-Fernández, 2024 | Long-Term Effects of a 6-Week Power-Based Resistance Training and Fast Walking Interval Training Program on Physical Function, Muscle Power, Disability, and Frailty in Pre-Frail and Frail Older Adults | Gerontology | Pre-frail/frail adults ≥75 years | Power RT + walking intervals; frailty/disability primary outcomes. | Wrong population (frail geriatric cohort) | https://doi.org/10.1159/000536363 |
| 10 | Chen, 2024 | Comparative Efficacy of Concurrent Training Types on Lower Limb Muscle Mass, Strength, and Explosive Force | Journal of Exercise Science & Fitness | Secondary synthesis of adult trials | Meta-analysis comparing concurrent training types; not a primary trial. | Secondary synthesis (meta-analysis) | https://doi.org/10.1016/j.jesf.2023.12.005 |
| 11 | Magalhães, 2019 | Effectiveness of high-intensity interval training combined with resistance training versus continuous moderate-intensity training combined with resistance training in patients with type 2 diabetes: A one-year randomized controlled trial | Diabetes, Obesity and Metabolism | Adults with type 2 diabetes | HIIT+RT vs MICT+RT; glycemic/cardiometabolic endpoints. “Part of the same 1-year RCT cohort as No.11–12 (secondary analysis / different endpoints).” | Wrong population (type 2 diabetes) | https://doi.org/10.1111/dom.13551 |
| 12 | Magalhães, 2019 | Effects of combined training with different intensities on vascular health in patients with type 2 diabetes: A one-year randomized controlled trial | Cardiovascular Diabetology | Adults with type 2 diabetes | Combined training with vascular outcomes (structure/function). “Part of the same 1-year RCT cohort as No.11–12 (secondary analysis / different endpoints).” | Wrong population; vascular outcomes | https://doi.org/10.1186/s12933-019-0840-2 |

| | | | | | | | |
|---|---|---|---|---|---|---|---|
| 13 | Magalhães, 2021 | Interindividual variability in fat mass response to a 1-year randomized controlled trial with different exercise intensities in type 2 diabetes: Implications on glycemic control and vascular function | Frontiers in Physiology | Adults with type 2 diabetes | Secondary analysis of RCT; responder variability; fat mass/vascular focus. "Part of the same 1-year RCT cohort as No.11–12 (secondary analysis / different endpoints)." | Secondary analysis; wrong population | https://doi.org/10.3389/fphys.2021.698971 |
| 14 | Nesti, 2020 | Type 2 diabetes and reduced exercise tolerance: A review of the literature through an integrated physiology approach | Cardiovascular Diabetology | Adults with type 2 diabetes | Narrative review; not an intervention trial. | Review article; no primary trial | https://doi.org/10.1186/s12933-020-01109-1 |
| 15 | de Mello, 2022 | Effect of high-intensity interval training protocols on VO2max and HbA1c level in people with type 2 diabetes: A systematic review and meta-analysis | Annals of Physical and Rehabilitation Medicine | Adults with type 2 diabetes | Systematic review/meta-analysis; HIIT-focused. | Systematic review/meta-analysis | https://doi.org/10.1016/j.rehab.2021.101586 |
| 16 | da Silva, 2019 | High-intensity interval training in patients with type 2 diabetes mellitus: A systematic review | Current Atherosclerosis Reports | Adults with type 2 diabetes | Systematic review of HIIT in T2D. | Systematic review | https://doi.org/10.1007/s11883-019-0767-9 |
| 17 | Peng, 2023 | The effect of low-volume high-intensity interval training on body composition and glycemic control in patients with type 2 diabetes: A systematic review and meta-analysis | Frontiers in Endocrinology | Adults with type 2 diabetes | Systematic review/meta-analysis of low-volume HIIT. | Systematic review/meta-analysis | https://doi.org/10.3389/fendo.2022.1098325 |
| 18 | De Nardi, 2018 | High-intensity interval training versus continuous training on physiological and metabolic variables in prediabetes and type 2 diabetes: A meta-analysis | Diabetes Research and Clinical Practice | Adults with prediabetes or type 2 diabetes | Meta-analysis comparing HIIT vs MICT. | Meta-analysis | https://doi.org/10.1016/j.diabres.2017.12.017 |
| 19 | Suryanegara, 2019 | High-intensity interval training protects the heart during increased metabolic demand in patients with type 2 diabetes: A randomised controlled trial | Acta Diabetologica | Adults with type 2 diabetes | HIIT trial with cardiac outcomes; no RT. | HIIT-only; wrong population | https://doi.org/10.1007/s00592-018-1245-5 |
| 20 | Ghardashi Afousi, 2018 | Improved brachial artery shear patterns and | Experimental Physiology | Adults with type 2 diabetes | HIIT vs comparator; vascular outcomes; no RT. | HIIT-only; wrong population | https://doi.org/10.1113/EP087005 |

| | | | | | | | |
|---|---|---|---|---|---|---|---|
| | | increased flow-mediated dilatation after low-volume high-intensity interval training in patients with type 2 diabetes | | | | | |
| 21 | Francois, 2018 | Cardiovascular benefits of combined interval training and post-exercise nutrition in patients with type 2 diabetes | Journal of Diabetes and its Complications | Adults with type 2 diabetes | Interval training plus nutrition; cardiometabolic outcomes. | No RT component; wrong population | https://doi.org/10.1016/j.jdiacomp.2017.10.002 |
| 22 | de Souza Mesquita, 2023 | Effect of high-intensity interval training on exercise capacity, blood pressure, and autonomic responses in patients with hypertension: A systematic review and meta-analysis | Sports Health | Adults with hypertension | Systematic review/meta-analysis of HIIT in hypertension. | Systematic review; wrong population | https://doi.org/10.1177/19417381221139343 |
| 23 | Leal, 2020 | Effectiveness of high-intensity interval training versus moderate-intensity continuous training in hypertensive patients: A systematic review and meta-analysis | Current Hypertension Reports | Adults with hypertension | Systematic review/meta-analysis of HIIT vs MICT. | Systematic review; wrong population | https://doi.org/10.1007/s11906-020-1030-z |
| 24 | Li, 2022 | Effects of high-intensity interval training versus moderate-intensity continuous training on blood pressure in patients with hypertension: A meta-analysis | Medicine (Baltimore) | Adults with hypertension | Meta-analysis on BP outcomes. | Meta-analysis; wrong population | https://doi.org/10.1097/MD.0000000000032246 |
| 25 | Romero-Vera, 2024 | Effects of high-intensity interval training on blood pressure levels in hypertensive patients: A systematic review and meta-analysis of randomized clinical trials | Life | Adults with hypertension | Systematic review/meta-analysis on BP reduction. | Systematic review; wrong population | https://doi.org/10.3390/life14121661 |
| 26 | Church, 2010 | Effects of Aerobic and Resistance Training on Hemoglobin A1c Levels in Patients With Type 2 Diabetes | JAMA | Adults with type 2 diabetes | RCT comparing aerobic, RT, combined training; HbA1c primary outcome. | Wrong population; outcomes not aligned | https://doi.org/10.1001/jama.2010.1710 |
| 27 | Sigal, 2007 | Effects of aerobic training, resistance training, or both on | Annals of Internal Medicine | Adults with type 2 diabetes | RCT comparing aerobic, RT, combined; glycemic outcomes. | Wrong population | https://doi.org/10.7326/0003-4819-147-6-200709180-00005 |

| | | | | | | | |
|---|---|---|---|---|---|---|---|
| | | glycemic control in type 2 diabetes | | | | | |
| 28 | Weston, 2014 | Effects of low-volume high-intensity interval training on fitness in adults with metabolic disease | Sports Medicine | Adults with cardiometabolic disease | Review/synthesis; not a primary trial. | Review article; no primary trial | https://doi.org/10.1007/s40279-014-0180-z |
| 29 | Ramos, 2015 | The impact of high-intensity interval training versus moderate-intensity continuous training on vascular function | Sports Medicine | Adults with cardiometabolic risk | Systematic review focusing on vascular outcomes. | Systematic review; vascular outcomes | https://doi.org/10.1007/s40279-015-0321-z |
| 30 | Liu, 2024 | Effects of aerobic exercise combined with resistance training on body composition and metabolic health in children and adolescents with overweight or obesity: systematic review and meta-analysis | Frontiers in public health | Children with overweight/obesity | Review; not a primary trial. | Review article; no primary trial | https://doi.org/10.3389/fpubh.2024.1409660 |
| 31 | Al-Mhanna, 2025 | Impact of high-intensity interval training on cardiometabolic health in patients with diabesity: a systematic review and meta-analysis of randomized controlled trials | Diabetology & metabolic syndrome | Adults with type 2 diabetes | Review; diabetes focus. | Review; wrong population | https://doi.org/10.1186/s13098-025-01974-4 |
| 32 | Ahmad, 2023 | Effects of low-versus high-volume high-intensity interval training on glycemic control and quality of life in obese women with type 2 diabetes. A randomized controlled trial | Journal of exercise science and fitness | Adults with type 2 diabetes | HIIT-only RCT; no RT. | HIIT-only; wrong population | https://doi.org/10.1016/j.jesf.2023.08.003 |
| 33 | Stensvold, 2010 | Strength training versus aerobic interval training to modify risk factors of metabolic syndrome | Journal of Applied Physiology | Adults with metabolic syndrome | RT vs aerobic interval; no concurrent arm. | Not concurrent HIIT+RT intervention | https://doi.org/10.1152/japplphysiol.00996.2009 |
| 34 | Tjønna, 2008 | Aerobic interval training versus continuous moderate exercise as a treatment for the metabolic syndrome | Circulation | Adults with metabolic syndrome | Aerobic interval vs continuous; no RT. | HIIT-only aerobic design | https://doi.org/10.1161/CIRCULATIONAHA.108.772822 |
| 35 | Karstoft, 2013 | The effects of free-living interval-walking training on glycemic control | Diabetes Care | Adults with type 2 diabetes | Interval walking vs continuous; no RT. | HIIT-style aerobic only; wrong population | https://doi.org/10.2337/dc12-0658 |

| | | | | | | | |
|---|---|---|---|---|---|---|---|
| 36 | Gibala, 2006 | Short-term sprint interval versus traditional endurance training: similar initial adaptations in human skeletal muscle | Journal of Physiology | Healthy young adults | Short-term SIT vs endurance; metabolic adaptations. | HIIT-only intervention; no RT | https://doi.org/10.1113/jphysiol.2006.112094 |
| 37 | Burgomaster, 2008 | Similar metabolic adaptations during exercise after low volume sprint interval and traditional endurance training in humans | Journal of Physiology | Healthy young adults | SIT vs endurance; metabolic outcomes. | HIIT-only intervention; no RT | https://doi.org/10.1113/jphysiol.2007.142109 |
| 38 | Little, 2010 | A practical model of low-volume high-intensity interval training induces mitochondrial biogenesis in human skeletal muscle | Journal of Physiology | Recreationally active adults | Low-volume HIIT; mitochondrial biogenesis markers. | HIIT-only; no RT | https://doi.org/10.1113/jphysiol.2009.181743 |
| 39 | Weston, 2014 | High-intensity interval training in patients with lifestyle-induced cardiometabolic disease: a systematic review and meta-analysis | British Journal of Sports Medicine | Adults with cardiometabolic disease | Systematic review/meta-analysis of HIIT. | Systematic review/meta-analysis | https://doi.org/10.1136/bjsports-2013-092576 |
| 40 | Milanović, 2015 | Effectiveness of high-intensity interval training (HIIT) and continuous endurance training for VO2max improvements: a systematic review and meta-analysis | Sports Medicine | Adults (mixed) | Meta-analysis of VO2max outcomes. | Systematic review/meta-analysis | https://doi.org/10.1007/s40279-015-0365-0 |
| 41 | Batacan, 2017 | Effects of high-intensity interval training on cardiometabolic health: a systematic review and meta-analysis of intervention studies | British Journal of Sports Medicine | Adults with cardiometabolic risk | Systematic review/meta-analysis. | Systematic review/meta-analysis | https://doi.org/10.1136/bjsports-2015-095841 |
| 42 | Wewege, 2017 | The effects of high-intensity interval training vs moderate-intensity continuous training on body composition in overweight and obese adults: a systematic review and meta-analysis | Obesity Reviews | Overweight/obese adults | Meta-analysis body composition. | Systematic review/meta-analysis | https://doi.org/10.1111/obr.12532 |
| 43 | Hwang, 2011 | Effect of aerobic interval training on exercise capacity and metabolic risk factors in people with cardiometabolic disorders: a meta-analysis | Journal of cardiopulmonary rehabilitation and prevention | Adults with metabolic disease/ heart disease | Meta-analysis; aerobic outcomes. | Meta-analysis | https://doi.org/10.1097/HCR.0b013e31822f16cb |

| | | | | | | | |
|---|---|---|---|---|---|---|---|
| 44 | MacInnis, 2017 | Physiological adaptations to interval training and the role of exercise intensity | Journal of Physiology | General/healthy | Narrative review. | Review article | https://doi.org/10.1113/JP273196 |
| 45 | Mølmen, 2025 | Effects of Exercise Training on Mitochondrial and Capillary Growth in Human Skeletal Muscle: A Systematic Review and Meta-Regression | Sports medicine (Auckland, N.Z.) | General/healthy | Systematic review and meta-regression. | Systematic review and meta-regression | https://doi.org/10.1113/JP275750 |
| 46 | Fyfe, 2014 | Interference between concurrent resistance and endurance exercise: molecular bases and the role of individual training variables | Sports medicine (Auckland, N.Z.) | Healthy and athletic | Review of interference mechanisms. | Review article | https://doi.org/10.1007/s40279-014-0162-1 |
| 47 | Coffey, 2017 | Concurrent exercise training: do opposites distract? | Journal of Physiology | General/healthy | Perspective/review on concurrent training. | Review article | https://doi.org/10.1113/JP272270 |
| 48 | Wilson, 2012 | Concurrent training: a meta-analysis examining interference of aerobic and resistance exercises | Journal of Strength and Conditioning Research | Mixed adults | Meta-analysis on interference. | Meta-analysis | https://doi.org/10.1519/JSC.0b013e31823a3e2d |
| 49 | Mateo-Gallego, 2022 | The effects of high-intensity interval training on glucose metabolism, cardiorespiratory fitness and weight control in subjects with diabetes: Systematic review a meta-analysis | Diabetes research and clinical practice | Adults with type 2 diabetes | Meta-analysis glycemic control. | Wrong population; meta-analysis | https://doi.org/10.1016/j.diabres.2022.109979 |
| 50 | Cornelissen, 2010 | Exercise training for blood pressure: a systematic review and meta-analysis | Journal of the American Heart Association | Healthy adults | Systematic review/meta-analysis of exercise and BP. | Wrong population; secondary synthesis | https://doi.org/10.1161/JAHA.112.004473 |
| 51 | Pedersen, 2009 | The diseasome of physical inactivity—and the role of myokines in muscle–fat cross talk | Journal of Physiology | General population | Mechanistic review; no trial. | Review article | https://doi.org/10.1113/jphysiol.2009.179515 |
| 52 | Egan, 2013 | Exercise metabolism and the molecular regulation of skeletal muscle adaptation | Cell Metabolism | General population | Review; no trial. | Review article | https://doi.org/10.1016/j.cmet.2012.12.012 |
| 53 | Bernedo, 2024 | An Exploratory Study on Whether the Interference Effect Occurs When High-Intensity Strength Training Is Performed | *Applied Sciences* | General population | Exercise intervention described as concurrent HIIT + resistance training; single-cohort / pre–post design without a parallel control group | Not controlled design | https://doi.org/10.3390/app14188447 |

| | | | | | | | |
|---|---|---|---|---|---|---|---|
| | | Prior to High-Intensity Interval Aerobic Training | | | | | |
| 54 | Feng, 2025 | Acute effects of high-intensity interval exercise and moderate-intensity continuous training on arterial stiffness and endothelial function in hypertension: A crossover trial | *Scientific Reports* | Participants with essential hypertension | HIIT and MICT sessions on a treadmill after a 1-week washout period | No musculoskeletal outcomes | https://doi.org/10.1038/s41598-025-21045-y |
| 55 | MacDonald, 2021 | Effects of interval training combined with muscle endurance training on cognitive function and cardiorespiratory fitness in patients with chronic lymphocytic leukemia: a randomized controlled trial | *Scientific Reports* | Chronic lymphocytic leukemia patients | Supervised interval training + muscle endurance / resistance-type training; randomized controlled trial in a clinical population | Wrong population | https://doi.org/10.1038/s41598-021-02352-6 |
| 56 | McGregor, 2016 | High-intensity interval training versus moderate-intensity steady-state training in UK cardiac rehabilitation programmes (HIIT or MISS UK): study protocol for a multicentre randomised controlled trial and economic evaluation | *BMJ Open* | Coronary heart disease patients (cardiac rehab) | Protocol paper comparing HIIT vs MISS (no concurrent HIIT+RT intervention implemented in the article) | Not concurrent HIIT+RT intervention | https://doi.org/10.1136/bmjopen-2016-012843 |
| 57 | Hoshino, 2023 | Abstracts presented at Stroke 2023 (includes HIIT-POST related preliminary abstract content) | *Int. J. Stroke* | Stroke-related conference abstracts | Abstract-supplement record; intervention/method details insufficient for study-level extraction and appraisal | Insufficient methodological detail | https://doi.org/10.1177/17474930231188838 |

Supplementary Table S2a provides a study-level log of all full-text records excluded during screening. For each report, we summarize the population, setting, intervention or study design, and indicate the primary exclusion reason, which corresponds to the aggregated categories reported in Supplementary Table S2 and Figure 1 (PRISMA 2020 flow diagram).
