# Supplementary material for: Concurrent HIIT and Resistance Training for Musculoskeletal Function: A Systematic Review of Neuromuscular, Morphological, and Performance Adaptations
Source: Life (Basel). 2026 Feb 27;16(3):381. doi: 10.3390/life16030381 (PMC13028498; doi:10.3390/life16030381)
Supplement: Supplementary file 1 [file life-16-00381-s001.zip › MDPI-LIFE-SR-Table S3.pdf]

Table S3. Adherence and safety reporting in included trials

Study (Ref.)	Population/setting	Duration of intervention	Adherence reporting*	Adverse events/safety
García Pinillos et al., 2019 [23]	Healthy older adults, community-dwelling	12 weeks	Supervised sessions; attendance reported as high across the program.	No exercise-related serious adverse events reported.
Wadsworth et al., 2022 [24]	Inactive aging women	10 weeks	Supervised concurrent sessions; adherence described as good based on session attendance.	No serious adverse events described; typical post-exercise soreness only.
Panissa et al., 2018 [25]	Recreationally trained young men	12 weeks	Training performed under supervision; most participants completed prescribed sessions.	No serious adverse events reported during strength or HIIT sessions.
Benítez-Flores et al., 2019 [26]	Recreationally active young men	2 weeks (6 sessions)	Short-term laboratory protocol with full supervision; near-complete completion of sessions.	No serious adverse events reported; sprint plus resistance training considered well tolerated.
Pugh et al., 2015 [27]	Healthy untrained young men	Single acute session (cross-over conditions)	All participants completed the experimental sessions; protocol fully supervised.	No adverse events reported during acute concurrent or single-mode sessions.
Campos Vázquez et al., 2015 [28]	Competitive youth soccer players	~7 weeks (preseason)	Team-based supervised training; high compliance with assigned sprint and strength sessions.	No exercise-related injuries beyond normal sport exposure reported.
Botonis et al., 2016 [29]	Elite male water-polo players	~8 weeks (in-season)	Supervised strength and interval endurance added to team practice; high attendance across sessions.	No serious adverse events reported; no withdrawals attributed to the intervention.
Wong et al., 2010 [30]	Professional male soccer players	~10 weeks (preseason)	Team-supervised conditioning; players generally completed planned concurrent sessions.	No intervention-related serious adverse events reported in the trial.
Müller et al., 2021 [31]	Healthy older men	12 weeks	Supervised 3×/week; adherence reported as high, with most participants completing planned sessions.	No serious exercise-related adverse events reported; protocol deemed safe for older men.

Study (Ref.)	Population/setting	Duration of intervention	Adherence reporting*	Adverse events/safety
Vlietstra et al., 2023 [32]	Middle-aged adults with low lean mass	12 weeks	Adherence defined as ≥90% attendance; proportion meeting the cut-off reported, with additional participants completing ≥70–89% of sessions.	No serious adverse events; minor musculoskeletal complaints monitored but not intervention-limiting.
Thomakos et al., 2023 [33]	Young competitive soccer players (preseason)	~7–8 weeks	Team-supervised programs; adherence high with most players completing prescribed sessions.	No serious adverse events reported; any missed sessions were mainly due to routine sport reasons.
Thomakos et al., 2024 [34]	Young soccer players (in-season)	6–8 weeks	In-season team training; adherence described as good across HIIT formats.	No intervention-related serious adverse events reported.
Robineau et al., 2017 [35]	Amateur rugby sevens players	8 weeks	Randomized into three groups; adherence sufficient for inclusion in final analyses, with some attrition mainly due to injury or missed sessions.	No serious adverse events uniquely attributed to the concurrent protocols; injuries comparable to usual training.
Leuchtmann et al., 2020 [36]	Healthy older men	12 weeks	Supervised training with good adherence; most participants completed HIIT and RT phases.	No exercise-related serious adverse events reported; biopsy procedures and training considered safe.
Kazior et al., 2016 [37]	Healthy young men	~7–8 weeks	Laboratory-based supervised programs; high completion of scheduled sessions.	No serious adverse events reported during endurance or strength sessions.
Spiliopoulou et al., 2021 [38]	Recreationally trained young men	8 weeks	Adherence to supervised power + HIIT sessions reported as high.	No serious adverse events described; minor transient soreness expected from training.
Sterczala et al., 2023 [39]	Recreationally active men and women, military-aged	12 weeks	Supervised training; adherence sufficient to detect improvements in occupational task performance.	No serious adverse events reported; training considered safe for men and women.
Sterczala et al., 2024 [40]	Recreationally active men and women	8–12 weeks	Supervised high-intensity low-volume protocol; adherence reported as high across the study.	No serious adverse events reported; concurrent program well tolerated.

* *Adherence reporting is summarized qualitatively based on session attendance/completion and the authors' description; most trials used supervised sessions with log-based monitoring rather than device-based tracking.*
