# Supplementary material for: Concurrent HIIT and Resistance Training for Musculoskeletal Function: A Systematic Review of Neuromuscular, Morphological, and Performance Adaptations
Source: Life (Basel). 2026 Feb 27;16(3):381. doi: 10.3390/life16030381 (PMC13028498; doi:10.3390/life16030381)
Supplement: Supplementary file 1 [file life-16-00381-s001.zip › MDPI-LIFE-SR-Table S4.pdf]

Table S4. Outcome domains and primary measures by study

Study (Ref)	Population	Strength measures	Power/speed measures	Morphology/tendon	Functional / task outcomes
García Pinillos 2019 [23]	Healthy older adults	Upper- and lower-body strength tests (e.g., chair stand, arm curl)	–	–	Mobility and balance tests (gait speed, postural sway)
Wadsworth 2022 [24]	Inactive older women	Dynamic strength of major muscle groups	–	–	Functional tests (chair rise, walking performance)
Panissa 2018 [25]	Recreational adults	1RM upper- and lower-body exercises; volume load	–	–	–
Benítez Flores 2019 [26]	Physically active adults	Multi-joint strength tests	Repeated sprint and high-intensity running performance	–	–
Pugh 2015 [27]	Young untrained men	Maximal strength of quadriceps	–	Muscle biopsy signaling markers	Molecular outcomes (mTOR, AMPK, related gene expression)
Campos Vázquez 2015 [28]	Young soccer players	1RM lower-body strength	CMJ, sprint performance, repeated sprint ability	–	–
Botonis 2016 [29]	Elite water polo players	Upper- and lower-body strength tests	Swimming sprint and repeated sprint performance	–	–
Wong 2010 [30]	Professional soccer players	Maximal strength of lower limbs	Sprint, agility, and repeated sprint tests	–	–
Müller 2021 [31]	Healthy older men	1RM leg press and other major muscle groups	Lower-limb power tests	–	Functional performance (sit-to-stand, walking capacity)
Vlietstra 2023 [32]	Middle-aged adults with low lean mass	1RM strength tests	–	Body composition (lean soft tissue)	Functional tests (mobility, daily activity performance)

Study (Ref)	Population	Strength measures	Power/speed measures	Morphology/tendon	Functional / task outcomes
Thomakos 2023 [33]	Young soccer players (pre-season)	Lower-body strength	CMJ height, short sprint times, intermittent endurance	–	–
Thomakos 2024 [34]	Young soccer players (in-season)	Isokinetic or dynamic strength indices	CMJ, sprint performance	–	–
Robineau 2017 [35]	Elite rugby sevens players	Resistance exercise performance	Sprint and repeated sprint tests	–	Rugby-specific performance outcomes
Leuchtmann 2020 [36]	Healthy older men	Maximal strength of lower limbs	–	Muscle capillarization and fiber characteristics (biopsy)	Functional performance (walking, stair climbing)
Kazior 2016 [37]	Young active men	Strength tests of major muscle groups	–	Fiber type–specific CSA and signaling markers (biopsy)	–
Spiliopoulou 2021 [38]	Young men	1RM lower-body and power-oriented lifts	CMJ, explosive performance indices	Muscle thickness and architecture (ultrasound)	–
Sterczala 2023 [39]	Men and women in military context	Strength tests (e.g., deadlift, upper-body strength)	–	–	Military occupational task performance (loaded march, obstacle tasks)
Sterczala 2024 [40]	Recreationally active men and women	Strength and explosive performance tests	Explosive tasks (e.g., jumps, IMTP metrics)	Muscle fiber CSA and related adaptations (biopsy)	–
