# Supplementary material for: Concurrent HIIT and Resistance Training for Musculoskeletal Function: A Systematic Review of Neuromuscular, Morphological, and Performance Adaptations
Source: Life (Basel). 2026 Feb 27;16(3):381. doi: 10.3390/life16030381 (PMC13028498; doi:10.3390/life16030381)
Supplement: Supplementary file 1 [file life-16-00381-s001.zip › MDPI-LIFE-SR-Table S5.pdf]

Table S5. PRISMA 2020 Checklist (with locations in the manuscript)

Section and Topic	Item	Checklist item	Location in manuscript
Title	1	Identify the report as a systematic review.	Title page: "Concurrent HIIT and Resistance Training for Musculoskeletal Function: A Systematic Review of Neuromuscular, Morphological, and Performance Adaptations." (p. 1)
Abstract	2	See the PRISMA 2020 for Abstracts checklist.	Structured abstract section, including background, objectives, methods (databases, eligibility criteria, number of trials), main results, and conclusions. (p. 1)
Rationale	3	Describe the rationale for the review in the context of what is already known.	Introduction, Sections 1.1–1.3 ("Musculoskeletal function and integrative assessment", "HIIT, resistance training, and neuromuscular adaptations", "Rationale for a systematic review on concurrent HIIT and resistance training"). (pp. 2-3)
Objectives	4	Provide an explicit statement of the objective(s) or question(s) the review addresses.	Introduction, Section 1.4 "Objectives". (p. 3)
Eligibility criteria	5	Specify the inclusion and exclusion criteria for the review and how studies were grouped for the syntheses.	Methods, Section 2.3 "Eligibility criteria" (PICO framework, including population, interventions, comparators, outcomes, study design, and additional exclusion criteria). (pp. 4-5)
Information sources	6	Specify all databases, registers, websites, organizations, reference lists, and other sources searched or consulted, and indicate the date when each was last searched.	Methods, Section 2.2 "Data Sources and Search Strategy" (four electronic databases, reference list screening, with searches conducted up to 30 November 2025). (p. 4)
Search strategy	7	Present the full search strategies for all databases, registers, and websites, including any filters and limits used.	Methods, Section 2.2 "Data Sources and Search Strategy" (narrative description in main text); full database search strings reported in Supplementary Table S1. (p. 4)
Selection process	8	Specify the methods used to decide whether a study met the inclusion criteria of the review, including how many reviewers screened each record and each report, whether they worked independently, and, if applicable, details of automation tools used.	Methods, Section 2.4 "Study selection" (two reviewers independently screened titles, abstracts, and full texts, handled duplicates, and resolved disagreements by discussion); flow of records depicted in Figure 1 (PRISMA flow diagram). (pp. 5-6)

Section and Topic	Item	Checklist item	Location in manuscript
Data collection process	9	Specify the methods used to collect data from reports, including how many reviewers collected data, whether they worked independently, any processes for obtaining or confirming data from study investigators, and, if applicable, details of automation tools used.	Methods, Section 2.5 “Data extraction” (one reviewer extracted data using a standardized form and a second reviewer verified all entries, with discrepancies resolved by discussion). (p. 6)
Data items	10a	List and define all outcomes for which data were sought. Specify whether all results that were compatible with each outcome domain in each study were sought, and if not, the methods used to decide which results to collect.	Methods, Section 2.5 “Data extraction” (musculoskeletal outcomes including maximal strength, explosive performance, neuromuscular activity, muscle morphology and architecture, tendon-related measures, and functional performance); details summarized in Supplementary Table S3. (p. 6)
Data items	10b	List and define all other variables for which data were sought, such as participant and intervention characteristics and funding sources. Describe any assumptions made about any missing or unclear information.	Methods, Section 2.5 “Data extraction” (sample size, age, sex, training status, population category, HIIT and resistance training structure, sequence and scheduling, adherence, and adverse events); adherence and safety information summarized in Supplementary Table S2. No formal assumptions for missing data are described. (p. 6)
Study risk of bias assessment	11	Specify the methods used to assess risk of bias in the included studies, including details of the tool(s) used, how many reviewers assessed each study and whether they worked independently, and, if applicable, details of automation tools used.	Methods, Section 2.7 “Risk of bias assessment” (Cochrane risk of bias tool for randomized trials, two reviewers assessing independently and resolving disagreements by discussion); results presented in Table 1 and Supplementary Table S4. (p. 7)
Effect measures	12	Specify, for each outcome, the effect measure(s) used in the synthesis or presentation of results.	Methods, Sections 2.5 and 2.6 and Results Sections 3.3–3.8. The review did not perform a formal meta-analysis. Effect measures followed those reported in the original studies (for example, changes in means, standard deviations, and reported effect sizes), which were summarized narratively. (pp. 6-7; pp. 19-22)
Synthesis methods	13a	Describe the processes used to decide which studies were eligible for each synthesis.	Methods, Sections 2.3 and 2.4 (eligibility criteria and selection process); Results, Sections 3.1 “Study Selection” and 3.2 “Characteristics of Included Studies”, which explain how 18 trials were included and grouped. (pp. 4-5; pp. 8-9)
Synthesis methods	13b	Describe any methods required to prepare the data for presentation or synthesis, such as handling of missing summary statistics or data conversions.	Methods, Section 2.5 “Data extraction” (extraction of pre to post means, standard deviations, and change scores where available, used for narrative synthesis and effect description). (p. 6)

Section and Topic	Item	Checklist item	Location in manuscript
Synthesis methods	13c	Describe any methods used to tabulate or visually display results of individual studies and syntheses.	Methods, Section 2.6 “Data synthesis”; Results tables and figures including Table 2 (study characteristics and main outcomes), Table 3 and Table 4 (summary of outcomes by population and training design), Figure 1 (flow diagram), and Supplementary Tables S2–S3. (p. 7; pp. 9-19; pp. 23-24; pp. 24-26; p. 6; pp. 6-7)
Synthesis methods	13d	Describe any methods used to synthesize results and provide a rationale for the choice of each method. State the methods used to explore possible causes of heterogeneity among study results.	Methods, Section 2.6 “Data synthesis” (due to substantial heterogeneity in training prescriptions, outcome measures, and reporting formats, the review used narrative synthesis instead of meta-analysis). Potential sources of heterogeneity are addressed qualitatively in the Results and Discussion. (p. 7)
Synthesis methods	13e	Describe any methods used to explore possible causes of heterogeneity among study results, for example subgroup analysis or meta-regression.	No formal statistical exploration of heterogeneity was conducted. Differences in outcomes across age groups, training status, and training structures are explored narratively in Results Sections 3.3–3.9 and summarized in Table 3 and Table 4. (pp. 19-23; pp. 23-24; pp. 24-26)
Synthesis methods	13f	Describe any sensitivity analyses conducted to assess robustness of the synthesized results.	No formal sensitivity analyses were performed (not applicable).
Reporting bias assessment	14	Describe any methods used to assess risk of bias due to missing results in a synthesis arising from reporting biases.	No formal method or tool for assessing reporting bias was applied. Potential limitations related to incomplete reporting are discussed in Discussion Section 4.8 “Limitations.” (pp. 30-31)
Certainty assessment	15	Describe any methods used to assess certainty or confidence in the body of evidence for an outcome.	No formal certainty assessment, such as GRADE, was conducted. The strength and limitations of the evidence are discussed narratively in the Discussion.
Study selection	16a	Describe the results of the search and selection process, from the number of records identified to the number of studies included in the review, ideally using a flow diagram.	Results, Section 3.1 “Study Selection” (numbers of records identified, screened, excluded, and included); Figure 1 (PRISMA 2020 flow diagram). (pp. 8-9; p. 6)
Study selection	16b	Cite studies that might appear to meet the inclusion criteria but which were excluded, and explain why they were excluded.	Supplementary Materials (exclusion list with reasons for exclusion), as noted in the Supplementary Materials section.
Study characteristics	17	Cite each included study and present its characteristics.	Results, Section 3.2 “Characteristics of Included Studies”; Table 2 (study design, participants, interventions, comparators, main outcomes); Tables 3 and 4; corresponding citations of included trials. (p. 9; pp. 9-19; pp. 23-24; pp. 24-26)

Section and Topic	Item	Checklist item	Location in manuscript
Risk of bias in studies	18	Present assessments of risk of bias for each included study.	Results and Methods, Section 2.7 "Risk of bias assessment"; Table 1 (risk of bias summary by domain); Supplementary Table S4 (item level risk of bias judgments). (p. 7; pp. 7-8; p. 8)
Results of individual studies	19	For all outcomes, present for each study: summary statistics for each group and an effect estimate and its precision, ideally using structured tables or plots.	Results, Sections 3.3–3.8 (outcome-specific subsections for strength, explosive performance, neuromuscular activity, morphology and architecture, tendon-related outcomes, and functional performance). Summary statistics and results are structured in Table 2, Table 3, Table 4, and Supplementary Table S3. (pp. 19-22; pp. 9-19; pp. 23-24; pp. 24-26; p. 7)
Results of syntheses	20a	For each synthesis, briefly summarize the characteristics and risk of bias among contributing studies.	Results, Sections 3.3–3.9 and Discussion Sections 4.1–4.7 (synthesis of adaptations in strength, power, neuromuscular function, morphology, tendon properties, and interference patterns in relation to risk of bias patterns summarized in Table 1 and Supplementary Table S4). (pp. 19-23; pp. 26-30)
Results of syntheses	20b	Present results of all statistical syntheses conducted. If meta-analyses were done, present effect estimates and confidence intervals and measures of statistical heterogeneity.	No statistical meta-analyses were conducted, so this item is not applicable.
Results of syntheses	20c	Present results of all investigations of possible causes of heterogeneity among study results.	No formal statistical investigation of heterogeneity was conducted. Narrative comparisons according to age group, training status, exercise sequencing, and scheduling are provided in Results Sections 3.3–3.9 and summarized in Tables 3 and 4. (pp. 19-23; pp. 23-24; pp. 24-26)
Results of syntheses	20d	Present results of all sensitivity analyses conducted to assess the robustness of the synthesized results.	No sensitivity analyses were performed (not applicable).
Reporting biases	21	Present assessments of risk of bias due to missing results arising from reporting biases for each synthesis assessed.	No formal reporting bias assessment was carried out. Potential selective reporting and publication bias issues are considered qualitatively in Discussion Section 4.8 "Limitations." (pp. 30-31)
Certainty of evidence	22	Present assessments of certainty or confidence in the body of evidence for each outcome assessed.	No formal certainty assessment tool was applied. Overall confidence in the evidence, practical implications, and research gaps are discussed in Discussion Sections 4.8 and 5 and in Section 6 "Conclusion." (pp. 30-31; pp. 31-32; p. 32)

Section and Topic	Item	Checklist item	Location in manuscript
Discussion	23a	Provide a general interpretation of the results in the context of other evidence.	Discussion, Sections 4.1–4.7 (integration of findings on strength, power, neuromuscular efficiency, structural adaptations, tendon responses, and interference considerations in relation to prior literature). (pp. 26-30)
Discussion	23b	Discuss any limitations of the evidence included in the review.	Discussion, Section 4.8 “Limitations” (small sample sizes, limited long term follow up, heterogeneity in training protocols and outcome measures, and incomplete reporting). (pp. 30-31)
Discussion	23c	Discuss any limitations of the review processes used.	Discussion, Section 4.8 “Limitations” (limitations in search coverage, lack of protocol registration, and absence of formal reporting bias and certainty assessments). (pp. 30-31)
Discussion	23d	Discuss implications of the results for practice, policy, and future research.	Discussion, Section 5 “Practical Implications” and Section 6 “Conclusion” (recommendations for programming concurrent HIIT and resistance training, guidance for practitioners, and directions for future studies). (pp. 31-32; p. 32)
Registration and protocol	24a	Provide registration information for the review, including register name and registration number, or state that the review was not registered.	Methods, Section 2.1 “Protocol and reporting” (the review protocol was not formally registered in a public registry). (pp. 3-4)
Registration and protocol	24b	Indicate where the review protocol can be accessed, or state that a protocol was not prepared.	Methods, Section 2.1 “Protocol and reporting” (no publicly accessible protocol is available). (pp. 3-4)
Support	25	Describe sources of financial or non financial support for the review, and the role of the funders.	“Funding” section (this research received no external funding and funders had no role). (p. 32)
Competing interests	26	Declare any competing interests of review authors.	“Conflicts of Interest” section (the authors declare no conflicts of interest). (p. 33)
Availability of data, code, and other materials	27	Report which of the following are publicly available and where they can be found: data collection forms, data extracted from included studies, data used for all analyses, analytic code, and any other materials used in the review.	“Data Availability Statement” (not applicable) and “Supplementary Materials” section (search strategies, adherence and safety summaries, outcome definitions, exclusion list, and detailed risk of bias assessments are provided in the supplements). (p. 33)
