# Supplementary material for: Concurrent HIIT and Resistance Training for Musculoskeletal Function: A Systematic Review of Neuromuscular, Morphological, and Performance Adaptations
Source: Life (Basel). 2026 Feb 27;16(3):381. doi: 10.3390/life16030381 (PMC13028498; doi:10.3390/life16030381)
Supplement: Supplementary file 1 [file life-16-00381-s001.zip › MDPI-LIFE-SR-Table S6.pdf]

Table S6. Detailed risk of bias judgments with rationales (RoB 2)

Study (Ref)	Randomization process	Deviations from intended interventions	Missing outcome data	Measurement of outcomes	Selection of reported results	Overall risk of bias
García Pinillos 2019 [23]	Low: random allocation described; groups comparable at baseline	Low: supervised sessions; co-interventions unlikely	Some concerns: attrition reported, but limited detail on reasons	Low: validated physical performance tests applied uniformly	Some concerns: no pre-specified analysis plan reported	Some concerns
Wadsworth 2022 [24]	High: single-group pre–post design with no random allocation or control group	High: intervention delivered as planned, but the single-group design means co-interventions and secular trends cannot be excluded	Some concerns: reasons for any missing follow-up data are not fully described	Some concerns: standard strength and function tests used without blinding; learning or familiarity effects cannot be ruled out	Some concerns: no registered protocol or pre-specified analysis plan available	High
Panissa 2018 [25]	Some concerns: randomization mentioned, but sequence generation not fully described	Low: adherence monitored; interventions delivered as planned	Low: very limited loss to follow-up	Low: 1RM and volume load measured with standard procedures	Some concerns: protocol registration not reported	Some concerns
Benítez Flores 2019 [26]	Some concerns: the allocation procedure is insufficiently detailed	Low: sprint and resistance sessions supervised; compliance high	Low: all participants included in final analyses	Low: performance and physiological outcomes measured with established protocols	Some concerns: unclear whether all pre-planned outcomes were reported	Some concerns
Pugh 2015 [27]	Low: randomized crossover design with counterbalanced order	Low: tightly controlled laboratory conditions; protocol adhered to in all trials	Low: complete dataset reported	Low: muscle biopsies and strength tests using standard techniques	Low: primary molecular outcomes clearly pre-specified in text	Some concerns
Campos Vázquez 2015 [28]	Some concerns: randomization stated, but concealment not described	Some concerns: potential differences in exposure to non-	Low: retention is high and similar across groups	Low: strength and sprint tests routinely used in soccer settings	Some concerns: no protocol; unclear if all outcomes reported	Some concerns

Study (Ref)	Randomization process	Deviations from intended interventions	Missing outcome data	Measurement of outcomes	Selection of reported results	Overall risk of bias
		study training are not fully controlled				
Botonis 2016 [29]	High: non-randomized allocation of players to groups within an in-season team setting	Some concerns: In-season team practices and competition exposure may have differed between groups	Low: most players completed follow-up testing and were included in analyses	Low: water polo-specific performance tests applied consistently across groups	Some concerns: lack of pre-registration; selective emphasis on significant findings cannot be excluded	High
Wong 2010 [30]	Some concerns: quasi-random allocation based on team logistics	Some concerns: Concurrent team training may have introduced an imbalance	Low: main outcomes available for almost all players	Low: validated field tests and strength measures	Some concerns: reporting focuses on primary performance outcomes; secondary endpoints less detailed	Some concerns
Müller 2021 [31]	Low: block randomization with clear description and concealment	Low: supervised center-based training; adherence closely tracked	Low: attrition low and reasons documented	Low: standard strength, power, and functional tests; assessors trained	Low: outcomes and analyses consistent with registered protocol	Some concerns
Vlietstra 2023 [32]	Low: computer-generated randomization; allocation concealed	Low: supervised exercise; contamination unlikely	Low: follow-up high with transparent reporting	Low: imaging and performance tests applied identically to both groups	Low: main and secondary outcomes reported as planned	Some concerns
Thomakos 2023 [33]	Some concerns: randomization process briefly described, concealment unclear	Low: teams trained under the same schedule; HIIT formats the main difference	Low: only a few dropouts; reasons reported	Low: CMJ, sprint, and Yo-Yo tests standard for youth soccer	Some concerns: no protocol; selective reporting cannot be ruled out	Some concerns

Study (Ref)	Randomization process	Deviations from intended interventions	Missing outcome data	Measurement of outcomes	Selection of reported results	Overall risk of bias
Thomakos 2024 [34]	Some concerns: allocation process not fully detailed	Low: in-season training controlled within the same club environment	Low: retention high across conditions	Low: neuromuscular and aerobic tests performed with consistent procedures	Some concerns: emphasis on primary performance variables; protocol not registered	Some concerns
Robineau 2017 [35]	Some concerns: randomization by training group; concealment not described	Some concerns: tournament schedule and travel may have affected exposure differently	Low: almost all players completed post-tests	Low: sprint and repeated sprint measures standard in rugby sevens	Some concerns: partial reporting of secondary variables	Some concerns
Leuchtmann 2020 [36]	Low: randomized parallel design described with adequate detail	Low: supervised training; co-interventions unlikely	Low: minimal attrition with reasons provided	Low: muscle biopsy and capillarization assessed with validated methods	Low: clear alignment between methods and reported outcomes	Some concerns
Kazior 2016 [37]	Low: randomization procedure described; groups comparable	Low: tightly supervised endurance and strength sessions	Low: complete or near-complete datasets	Low: muscle biopsy and strength measures standardized	Some concerns: absence of protocol registration	Some concerns
Spiliopoulou 2021 [38]	Some concerns: randomization mentioned but not fully detailed	Low: supervised power training and HIIT cycling; adherence high	Low: nearly all participants completed testing	Low: ultrasound and performance tests applied consistently	Some concerns: selective reporting cannot be excluded	Some concerns
Sterczala 2023 [39]	Low: randomized parallel-group design clearly reported	Low: military-style training delivered under controlled conditions	Low: attrition low; reasons described	Low: occupational task tests and strength assessments objectively measured	Low: primary and secondary outcomes reported in full	Some concerns
Sterczala 2024 [40]	Low: randomization process transparent; concealment adequate	Low: supervised concurrent training; protocol deviations minimal	Low: very few missing data points with explanations	Low: muscle adaptations and performance outcomes measured with standard laboratory techniques	Low: outcomes align with methods and planned analyses	Some concerns

Note: This table provides full domain-level judgments and justifications for each comparative trial. RoB 2 was applied to randomized, crossover, and cluster-randomized studies, while ROBINS-I was used for the nonrandomized comparative study. The pre–post single-group study by Wadsworth et al. 2022 [24] was not appraised with RoB 2 or ROBINS-I. It was judged to have a high overall risk of bias due to the absence of a control group, lack of randomization, and susceptibility to confounding, maturation, and other time-related effects that limit causal attribution of pre–post changes to the intervention.
